# Various optimization algorithms for efficient placement and sizing of photovoltaic distributed generations in different networks

**DOI:** 10.1371/journal.pone.0319422

**Published:** 2025-04-02

**Authors:** Ahmed A. Zaki Diab, Fayza S. Mahmoud, Hamdy M. Sultan, Abou-Hashema M. El-Sayed, Mohamed A. Ismeil, Omar Makram Kamel

**Affiliations:** 1 Department of Electrical Engineering, Faculty of Engineering, Minia University, Minia, Egypt; 2 Minia National University, Minia, Egypt; 3 Electrical Engineering Department, Faculty of Engineering, King Khalid University, Abha, Saudi Arabia; 4 Electrical and Computer Engineering Department, Minia Higher Institute of Engineering and Technology, New El-Minya, Minia, Egypt; Aalto University, FINLAND

## Abstract

Recent research has concentrated on emphasizing the significance of incorporating renewable distributed generations (RDGs), like photovoltaic (PV) and wind turbines (WTs), into the distribution system to address issues related to distributed generation (DG) allocation. The key implications of integrating RDGs include the improvement of voltage profiles and the minimization of power losses. Various optimization techniques, namely Salp Swarm Algorithm (SSA), Marine Predictor Algorithm (MPA), Grey Wolf Optimizer (GWO), Improved Grey Wolf Optimizer (IGWO), and Seagull Optimization Algorithm (SOA), have been applied to achieve optimal allocation and sizing of RDGs in radial distributed systems (RDS). The present paper is structured in two phases. In the initial phase, the Loss Sensitivity Factor (LSF) is employed to identify the most suitable nodes for integrating RDGs. In the second phase, within the selected candidate nodes from the first phase, the optimal location and capacity of RDGs are determined. Additionally, a comprehensive comparison of the proposed optimization methods is conducted to select the most effective solutions for the allocation of units of RDGs. The efficacy of the utilized techniques is validated through testing on two distinct networks, namely the IEEE 33 and 69 buses RDS in MATLAB, with attainments compared against other techniques. Moreover, a larger RDS system of 118- bus IEEE system has been considered in order to enhance its power quality indices. Moreover, a real case of study from Egypt of 15 bus has been considered and evaluated with considering the applied techniques. The results show the enhancement of the voltage profile and decreasing the power losses of the tested system with the DG systems with the superiority of the MPA and SSA algorithms.

## I. Introduction

Over the past few decades, the integration of DGs into distribution networks has played a pivotal role in the planning of distribution networks, owing to its myriad technical, environmental, and economic advantages [1,2]. DGs are essentially small-scale power plants that are either connected to the grid or strategically installed in proximity to end-users. This technology encompasses three main types: renewable DGs such as wind, photovoltaic (PV), fuel cells, biomass, etc., conventional DGs, and hybrid systems that combine both. The current study landscape is increasingly focused on the installation of renewable distributed generations into radial systems, with a particular emphasis on PV and WT systems. This emphasis stems from the quest to harness cleaner energy sources, capitalizing on the inherent availability of solar and wind energy in nature [3–5]. This growing interest underscores the commitment to advancing sustainable energy solutions and signifies a shift towards cleaner and more environmentally friendly power generation methodologies.

On the other hand, RDGs have numerous technical merits like decreasing power loss, improving voltage profile, and finally, improving the thoroughness and effectiveness of the distribution network. Also, the environmental and economical benefits are represented in the decrease of the consumption of fuels, electricity prices, and costs for transmission and distribution of electric energy [6–8]. So, the integration of RDGs represents an excellent solution to ameliorate the power quality indicators and power system stability. But such ultimate advantages are only attained when RDGs are established in suitable places and with the proper Rating, otherwise incorrect location and capacity of RDGs may lead to more technical troubles. Therefore, optimal placing and sizing of RDG is a complex engineering issue that is solved by several methods.

### A. Literature review

In recent times, various optimization techniques have proven effective in addressing the challenges related to the allocation and sizing of Renewable Distributed Generations (RDGs). These optimization algorithms can be broadly categorized into three categories: analytical methods, meta-heuristic methods, and hybrid optimization methods [9]. Analytical methods include iterative-analytical methods [10], analytical technique [11], and efficient analytical(EA) technique [12]. Despite these efforts, many attempts have fallen short in accurately identifying the optimal allocation of multiple RDGs, prompting researchers to explore more robust approaches [13]. In response to these challenges, meta-heuristic optimization techniques have emerged as a promising solution capable of overcoming the complexities of achieving the optimum sizing of RDGs and yielding high-quality attainments. This shift toward meta-heuristic approaches reflects a recognition of their ability to navigate the intricate optimization landscape and provide more effective solutions for the integration of RDGs into distribution systems.

Thus, meta-heuristic algorithms are employed to address optimization problems, with notable techniques including [14], artificial bee colony method (ABC) [15], plant propagation algorithm (PPA) [16], Manta Ray Foraging optimization algorithm (MRFO) [17], honey badger algorithm (HBA) [18], multileader particle swarm optimization (MLPSO) [19], capuchin search algorithm (CapSA) [20], honey bee mating optimization (HBMO) [21], whale optimization algorithm (WOA) [22], Equilibrium optimization (EO) [23], Harris hawks optimizer (HHO) [24], Black Widow Optimizer (BWO) [5], Sine Cosine Algorithm (SCA) [25], Multi Verse Optimization (MVO) [26], Tunicate Swarm Algorithm (TSA) [27], harmony search (HS) [28,29], modified crow search (MCS) [30], adaptive cuckoo search (ACS) [31], Modified Gradient-Based Optimization (MGBO) [32], fireworks algorithm (FA) [33], coyote algorithm [34], modified plant growth simulation (MPGS) [35], the Jellyfish search algorithm (JS) [36], improved African vultures optimizer (IAVO) [37], Artificial Gorilla Troops Optimizer (AGTO) [38], bald eagle search algorithm [39], and artificial ecosystem-based optimization (AEO) [40]. Hybrid methodologies have been devised to address the limitations encountered in both analytical and meta-heuristic approaches. A notable illustration of hybrid methodologies involves the simultaneous application of the Grasshopper Optimization Algorithm (GOA) and Cuckoo Search (CS) [41]. This collaborative approach harnesses the strengths of both algorithms, synergistically combining their unique attributes to enhance the overall optimization process. By integrating analytical and meta-heuristic elements, hybrid methodologies aim to leverage the complementary strengths of different optimization techniques, thereby overcoming challenges more effectively and achieving superior results in the allocation and sizing of renewable distributed generations. Also, improved PSO and gravitational search algorithm (WIPSO–GSA) is proposed in [42], hybrid genetic particle swarm optimization [43], and hybrid PSOGSA optimization algorithm [44].

Author in [45] implemented the enhanced sunflower optimization (ESFO) for optimal placement and capacity of RDGs in the distribution networks while the objective function is formulated to reduce active power losses and enhance the power quality indicators of the distribution network. In [46] moth-flame optimization (MFO) and hybrid algorithm incorporating particle swarm optimization and gravitational search algorithm(PSOGSA) are employed to select the optimum place and sizing of RDGs for improving voltage shape and voltage stability index while reducing power losses and operating cost of the system. Moreover, a comprehensive comparison was employed with recent optimization methods to prove the supremacy of the suggested technique over the other algorithms. the critical buses of RDGs is selected by the loss sensitivity factors (LSFs), Then the capacity of RDGs is achieved using a bacterial foraging optimization algorithm(BFOA) [47]. In this research, the decrease of active power losses and improvement of voltage profile is considered as the main task to integrate the RDGs into distribution systems. The optimum size of RDGs (PV and WT) and their place can be evaluated by backtracking search optimization algorithm (BSOA). The scope of this work is proposed to reduce the system losses and enhance the voltage shape of the buses in radial distribution network [48]. A hybrid methodology for best sizing and allocation of RDGs with different types is presented in this work for decreasing the line losses and enhancing the voltage shape [49]. In [50] a new multi-objective sine cosine algorithm(MOSCA) was utilized for identifying the optimum place of RDG units in IEEE 33 and 69-bus standard networks. This method is applied for minimizing the power losses, minimizing yearly energy production costs, decreasing greenhouse gases emissions, and upgrading the voltage stability index.

According to previous review the following notes is obtained. The proper allocation of DGs into conventional power systems has positive impacts. These impacts can be concluded as following: decreasing the net investment and operating costs of conventional generators, Increasing the power system stability and reliability, and final reducing the greenhouse emissions from conventional power stations [51]. Finding the proper position of DGs is considered to be a complicated problem that depends on a multiobjective function. This function constrained by limits of system dynamic stability and economic point of view [52]. There are four primary methods deployed to deal with this problem. The first two methods are classical techniques, Sensitivity analysis-based approaches, Metaheuristic-based approaches, and Hybrid of sensitivity analysis and classic/metaheuristic-based approaches. These methods typically have preconditions like convexity, linearity, and continuity of objective functions, which are not met in practice, and so suffer from a lack of flexibility. Also These two methods the degree of optimization of the solutions reached is uncertain, with low processing time [53]. The third method is Metaheuristic-based approaches. They are the most widely used methods for resolving the DG allocation problem because they are effective. However and they might converge to the local optimal state rather than the global optimum solution [54]. Hybrid of sensitivity analysis and classic/metaheuristic-based approaches is the fourth category. These methods provides faster solutions without getting stack to the local optimum points without using mixed integer optimization equations [55].

### B. Paper contribution and organization

The optimization methods discussed in the literature are among the most advanced and efficient, offering significant benefits. However, they also exhibit certain limitations, such as slow convergence rates and a tendency to get trapped in local optima. These drawbacks highlight gaps in achieving optimal solutions, underscoring the need for more effective approaches capable of delivering global or near-global solutions.

A review of the literature further indicates that most research efforts have been directed toward small- to medium-sized distribution systems, where distributed generators (DGs) are predominantly employed to reduce power losses. In contrast, this study broadens the scope by utilizing DGs to address multiple objectives, including mitigating real power losses, minimizing voltage variations, and reducing overall costs.

To achieve these goals, this manuscript employs five advanced optimization algorithms: SSA, MPA, GWO, IGWO, and SOA. These population-based metaheuristic strategies are known for their simplicity, flexibility, and adaptability, offering distinct advantages over traditional methods. The selected algorithms demonstrate robust global search capabilities and efficient convergence speeds when applied to virtual datasets. In this research, they have been successfully implemented to determine the optimal placement of DGs in radial distribution networks, effectively achieving the objectives of reducing real power losses, minimizing voltage variations, and lowering costs.

In the current study, the recommended approaches have been implemented across diverse distribution grids, including the 33-bus and 69-bus configurations, to determine the optimal location in Radial Distribution Networks (RDN). The forward–backward sweep (BFS) is employed for its notable advantages, including high convergence, precision, and flexibility [56,57].

The suggested optimization techniques are specifically incorporated for the injection of real power from Distributed Generations (DG), particularly photovoltaic (PV) systems. The application of the BFS algorithm in RDN enhances the efficiency of the optimization process. Furthermore, the inclusion of the Loss Sensitivity Factor (LSF) is integral to the methodology, aiding in the identification of nodes with the most significant reduction in losses upon DG integration. Additionally, the suggested techniques are utilized to estimate the optimal allocation of renewable distributed generations in the distribution grid, along with determining their appropriate sizes. To validate the efficacy of the proposed methodologies, comprehensive comparisons are conducted with other methods, and the simulations are executed using the MATLAB environment. The obtained results provide insights into the performance of the suggested techniques, offering a basis for evaluating their effectiveness in optimizing the integration of RDGs into distribution networks.

The outlines of this paper are planned as follows: the mathematical modelling, formulation of the fitness function and constraints are discussed in section Problem statement, the explanation of the suggested optimization methods is described in section Optimization Methods. The simulation attainments are presented in section Simulation Results. Moreover, the Statistical Results section presents the analysis of the statistical results. Finally, the Conclusion section summarizes the major conclusion and recommendations for future work.

## II. Problem statement

### A. Power flow calculations

**Fig 1:**
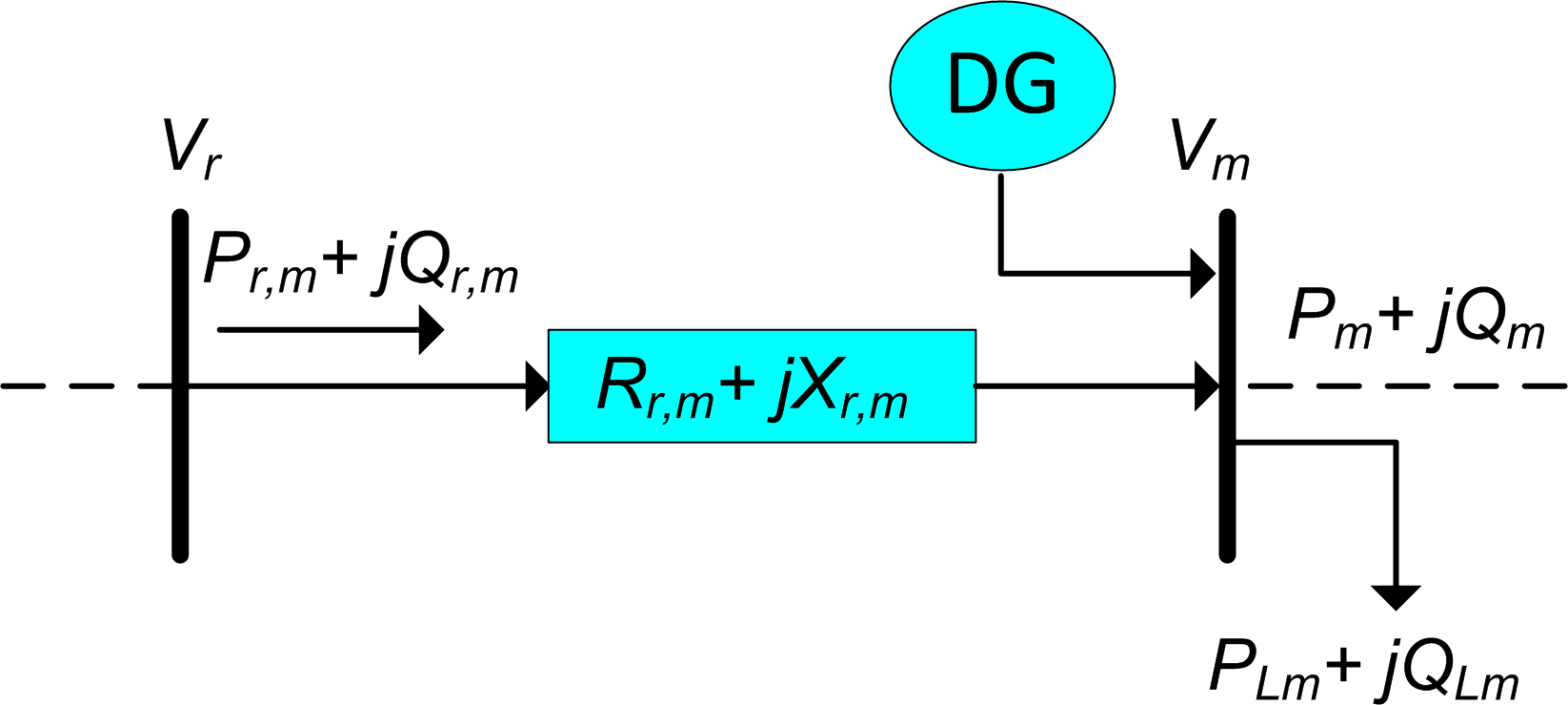
Single line diagram of RDN.

The primary objective of this work is to identify the optimal places and capacities of renewable distributed generations, PV systems, within radial networks with the aim of minimizing system losses while adhering to various constraints. The combined power derived from the infinite bus and the cumulative generation from nonconventional sources in the system must equal the load consumption and power losses in the network. In Fig 1, the diagram illustrates a radial network with a line denoted as n_mr connecting two busbars “r” and “m.”

To address load flow issues in radial distributors, the Backward/Forward Sweep (BFS) methodology is employed, which comprises three steps: (i) Backward Sweep, (ii) Forward Sweep, and (iii) nodal analysis [58]. Convergence is achieved when the maximum voltage mismatch is below the epsilon tolerance (ε_t), set at 0.00001. Once load flow converges, the identification of reactive and real power loss in the network becomes straightforward. The BFS power flow calculations involve estimating backward sweep direction using the formula presented in equation (1). This iterative process ensures an accurate assessment of the system’s power flow characteristics, facilitating the identification of optimal RDG locations and capacities in radial networks.


$${\text{P}}_{\text{r},\text{m}}={P^{\prime }}_{\text{m}}+{\text{R}}_{\text{r},\text{m}}\frac{\left({P^{\prime }}_{m}^{2}+{Q^{\prime }}_{m}^{2}\right)}{{\text{V}}_{\text{m}}^{2}}$$ (1)

$${\text{Q}}_{\text{r},\text{m}}={Q^{\prime }}_{\text{m}}+{\text{X}}_{\text{r},\text{m}}\frac{\left({P^{\prime }}_{m}^{2}+{Q^{\prime }}_{m}^{2}\right)}{{\text{V}}_{\text{m}}^{2}}$$ (2)

Here ${P^{\prime }}_{\text{m}}={\text{P}}_{\text{m}}+{\text{P}}_{\text{Lm}}$ and ${Q^{\prime }}_{\text{m}}={\text{Q}}_{\text{m}}+{\text{Q}}_{\text{Lm}}$, ${\text{P}}_{\text{Lm}}$ and ${\text{Q}}_{\text{lm}}$ denote the real and reactive power drawn by the consumer of bus “m”.

During the forward sweep phase, the determination of voltage amplitude and phase angle at individual buses is conducted. In this context, the notation ${\text{V}}_{\text{rr}}$ is utilized to represent voltage of bus ‘r,’ while ${\text{V}}_{\text{mm}}$ corresponds to voltage of bus ‘m.’ These expressions serve as descriptors for the voltage characteristics at specific nodes within the radial distribution system. This step in the forward sweep process is crucial for assessing the voltage profiles across different network nodes. The detailed estimation of voltage amplitudes and angles provides valuable insights into the electrical behavior of the system. Such insights are instrumental in comprehensively understanding the distribution network’s performance, thereby contributing to the identification of optimal locations and capacities for integrating RDGs like PV systems.

In accordance with the third step, which involves nodal current analysis, the current flowing through the line with impedance, $Z_{rm}=R_{rm}+jX_{rm}$ positioned between bus “r” and bus “m,” can be calculated as follows:


$${\text{I}}_{\text{r},\text{m}}=\frac{\left({\text{V}}_{\text{r}}\text{r}-{\text{V}}_{\text{m}}\text{m}\right)}{{\text{R}}_{\text{r},\text{m}}+{\text{jX}}_{\text{r},\text{m}}}$$ (3)

$${\text{I}}_{\text{r},\text{m}}=\frac{\left({\text{P}}_{\text{r}}-{\text{jQ}}_{\text{r}}\right)}{{\text{V}}_{\text{r}}-\text{r}}$$ (4)

The estimated voltage at bus “m” is formulated as given in (5),


$${\text{V}}_{\text{m}}={\left[{\text{V}}_{\text{r}}^{2}-2\left({\text{P}}_{\text{r}}{\text{R}}_{\text{r},\text{m}}+{\text{jQ}}_{\text{r}}{\text{X}}_{\text{r},\text{m}}\right)+\left({\text{R}}_{\text{r,m}}^{2}+{\text{X}}_{\text{r,m}}^{2}\right).\frac{\left({\text{P}}_{\text{r}}^{2}+{\text{jQ}}_{\text{r}}^{2}\right)}{{\text{V}}_{\text{r}}^{2}}\right]}^{\frac{1}{2}}$$ (5)

Here, ${\text{P}}_{\text{r}}$ and ${\text{Q}}_{\text{r}}$ represent the real and reactive power flow through the section connecting buses r and m. ${\text{R}}_{\text{r},\text{m}}$ and ${\text{X}}_{\text{r},\text{m}}$ refer to the resistance and inductive reactance of the section from node “r” to node “m.” Additionally, ${\text{V}}_{\text{r}}$ and ${\text{V}}_{\text{m}}$ denote the voltage at buses “ *r*” and “*m*”.

Then, the losses of real and reactive power in the section between bus “r” and bus “m” before installation of RDG can be formulated from equations below:


$${\text{P}}_{\text{loss}\left(\text{r},\text{m}\right)}={\text{R}}_{\text{rm}}\frac{\left({\text{P}}_{\text{rm}}^{2}+{\text{jQ}}_{\text{rm}}^{2}\right)}{{\text{V}}_{\text{r}}^{2}}$$ (6)

$${\text{Q}}_{\text{loss}\left(\text{r},\text{m}\right)}={\text{X}}_{\text{rm}}\frac{\left({\text{P}}_{\text{rm}}^{2}+{\text{jQ}}_{\text{rm}}^{2}\right)}{{\text{V}}_{\text{r}}^{2}}$$ (7)

The total real power losses in RDS is expressed as follow:


$${\text{P}}_{\text{loss}\left(\text{r},\text{m}\right)}^{\text{T}}=\sum_{\text{m}=1}^{\text{n}}{\text{P}}_{\text{loss}\left(\text{rm}\right)}$$ (8)

where, ‘ ${\text{n}}_{\text{br}}$ ’ indicates the number of the branches, r = 1: n, and ‘n’ denotes to the number of buses.

### B. Determination of power losses with RDG

Installation of RDG to the radial distribution systems changes the direction of power flow in the lines of the system. Therefore, in this case, the active and reactive power transfer between nodes “r “and “m” is the case of integrating photovoltaic systems (PVs) is calculated as follows:


$${\text{P}}_{\text{r},\text{m}}={P^{\prime }}_{\text{m}}+{\text{R}}_{\text{r},\text{m}}\frac{\left({P^{\prime }}_{m}^{2}+{Q^{\prime }}_{m}^{2}\right)}{{\text{V}}_{\text{m}}^{2}}-{\text{P}}_{\text{DG},\text{m}}$$ (9)

$${\text{Q}}_{\text{r},\text{m}}={Q^{\prime }}_{\text{m}}+{\text{X}}_{\text{r},\text{m}}\frac{\left({P^{\prime }}_{m}^{2}+{Q^{\prime }}_{m}^{2}\right)}{{\text{V}}_{\text{m}}^{2}}$$ (10)

Thus, the real power losses occurred in the line from bus “r” to bus “m” can be calculated by:


$${\text{P}}_{\text{RDG},\text{loss}\left(\text{rm}\right)}={\text{R}}_{\text{rm}}\frac{\left({\text{P}}_{\text{RDG}\left(\text{rm}\right)}^{2}+{\text{jQ}}_{\text{RDG}\left(\text{rm}\right)}^{2}\right)}{{\text{V}}_{r}^{2}}$$ (11)

And the overall active power losses after installation of RDG units can be determined as:


$${\text{P}}_{\text{RDG},\text{loss}\left(\text{r},\text{m}\right)}^{\text{T}}=\sum_{\text{m}=1}^{\text{n}}{\text{P}}_{\text{RDG},\text{loss}\left(\text{rm}\right)}$$ (12)

### C. Power losses index

It is known as the ratio of power loss in the case of integrating RDGs to losses in the base case, and is determined by:


$$\Delta {\text{P}}_{\text{l},\text{DG}}=\frac{{\text{P}}_{\text{RDG},\text{loss}\left(\text{r},\text{m}\right)}^{\text{T}}}{{\text{P}}_{\text{loss}\left(\text{r},\text{m}\right)}^{\text{T}}}$$ (13)

### D. Voltage stability index

Voltage stability is a critical aspect of power system operation, and it becomes even more crucial in distribution networks when integrating distributed generation (DG) units. Voltage stability index (VSI) is a factor utilized to evaluate the voltage stability in a power system, and it can be particularly relevant in the context of DG allocation in distribution networks. The VSI is a quantitative measure that indicates the proximity of the power system to a voltage collapse or instability condition. It is often computed based on system parameters such as bus voltages, power injections, and line impedances. One of the main targets of using the voltage stability index is to evaluate the security level [59] that can be evaluated as follows:


$${\text{VSI}}_{\text{m}}=[{\text{V}}_{r}^{4}-4\text{*}[{\text{P}}_{\text{m},\text{eff}}{\text{*X}}_{\text{r},\text{m}}-{\text{Q}}_{\text{m},\text{eff}}{\text{*R}}_{\text{r},\text{m}}{]}^{2}-4\text{*}\left[{\text{P}}_{\text{m},\text{eff}}{\text{*R}}_{\text{rm}}-{\text{Q}}_{\text{m},\text{eff}}{\text{*X}}_{\text{rm}}\right]{\text{*V}}_{r}^{2}$$ (14)

### E. Voltage deviation index

The voltage deviation index is another important metric in the context of DG allocation in distribution networks. Voltage deviation refers to the variation of bus voltages from their nominal values, and the voltage deviation index quantifies this variation. In the context of DG allocation, managing voltage deviation becomes crucial to ensure the quality and stability of the distribution network. The voltage deviation index $\Delta {\text{V}}_{\text{Dev}}$ [47] is presented as follows:


$$\Delta {\text{V}}_{\text{Dev}}=\text{max}\left(\frac{{\text{V}}_{1}-{\text{V}}_{\text{r}}}{{\text{V}}_{1}}\right),\text{r}=1,2,\ldots \ldots \ldots \ldots ,\text{n }$$ (15)

### F. Total operational cost (TOC)

The major task of implementing the proposed system is to decrease power losses, which minimizes the total operating cost. The integration of RDGs helps to reduce the energy drawn from the utility grid and minimizes the overall energy cost. The reduction of the operational cost is treated as a merit of RDGs allocation in the RDN [31]. The total operation cost is categorized into two parts, the first is regarded as the real power drawn from the network. The next is represented as the overall operation cost after installing RDGs. The total operational cost (TOC) is calculated as:


$$\text{TOC}=\left({\text{κ}}_{1}{\text{*P}}_{\text{DG},\text{Tloss}}\right)+\left({\text{κ}}_{2}{\text{*P}}_{\text{T},\text{DG}}\right)$$ (16)

where, ${\text{κ}}_{1}$ and ${\text{κ}}_{2}$ denote to cost parameters of active power fed by grid and renewable generation ($/kW). ${\text{P}}_{\text{T},\text{DG}}$ denotes overall real power supplied by RDGs. The ΔOC is reduced as:


$$\Delta \text{OC}=\frac{\text{TOC}}{{\text{κ}}_{2}{\text{*P}}_{\text{T},\text{DG}}^{\text{max}}}$$ (17)

### G. Objective function

In the present study, the objective function (OF) designed for the optimal allocation of renewable distributed generations is structured as a multi-objective function. This function aims to concurrently minimize the overall losses, voltage deviations, and overall operation cost of the radial distribution system, while simultaneously raising the voltage shape and improving VSI. The formulation of the OF is assessed through equation (18):


$$\min\left(\text{OF}\right)=\lambda_{1}\Delta {\text{P}}_{\text{l},\text{DG}}+\lambda_{2}\Delta {\text{V}}_{\text{Dev}}+\lambda_{3}\Delta \text{OC}$$ (18)

where, ${\text{λ}}_{1}$, ${\text{λ}}_{2}$, and ${\text{λ}}_{3}$ denote the weighting factors of the objective function.

### H. Constraints

#### 1) Equality constraints.

Power balance: One of the fundamental equality constraints is the power balance equation, which ensures that the total power supplied into the system equals the overall power consumed. The total real power flow of the RDG can be summarized as:


$${\text{P}}_{\text{slack}}+\sum_{\text{r}=1}^{{\text{n}}_{\text{DG}}}{\text{P}}_{\text{DG},\left(\text{m}\right)}=\sum_{\text{r}=1}^{\text{n}}{\text{P}}_{\text{L}\left(\text{m}\right)}+\sum_{\text{m}=1}^{{\text{n}}_{\text{br}}}{\text{P}}_{\text{loss}\left(\text{rm}\right)}$$ (19)

#### 2) Inequality constraints.

Voltage magnitude limits: the voltage amplitude at each node must occur between the lower (${\text{V}}_{\text{min}}=0.95)$ and upper (${\text{V}}_{\text{max}}=1.05$) boundaries.


$${\text{V}}_{\text{min}}{\text{<V}}_{\text{r}}{\text{<V}}_{\text{max}}$$ (20)

Voltage drop limits: the voltage drop limits can are defined as:


$${\text{V}}_{1}-{\text{V}}_{\text{r}}\leq \Delta {\text{V}}^{\text{Dev max}}$$ (21)

DG sizing/ capacity limits: the total active power generated from RDGs is subjected to ${\text{P}}_{\text{T},\text{DG}}^{\text{min}}$ and ${\text{P}}_{\text{T},\text{DG}}^{\text{max}}$ as given below [31]:


$${\text{P}}_{\text{T},\text{DG}}^{\text{min}}{\text{<P}}_{\text{T},\text{DG}}{\text{<P}}_{\text{T},\text{DG}}^{\text{max}}$$ (22)

The apparent power flowing via the line ${\text{S}}_{\text{r}}$ is constrained by its max allowable limit ${\text{S}}_{\text{r}}^{\text{rated}}$ by the below equation,


$${\text{S}}_{\text{r}}<{\text{S}}_{\text{r}}^{\text{rated}}$$ (23)

### I. Sensitivity factors analysis

#### 1) Loss Sensitivity Factors (LSFS).

Loss Sensitivity Factors (LSFs) are parameters used in power system analysis to quantify the sensitivity of power losses in the network to variations in active power (real power) injections at different buses. LSFs provide insights into how the distribution of power generation and consumption affects system losses. These factors are particularly useful in optimizing power flow and determining the impact of changes in the power system configuration on overall losses. The LSF for a particular bus is defined as the partial derivative of total system losses with respect to the active power supply at that bus, while keeping other injections constant. The LSFs are used in this study to specify the most suitable candidate nodes for RDGs integration using the load flow algorithm. The achievements of LSF are organized in descending order for overall the branches of the network. According to Fig 1, $P_{m,eff}+jQ_{m,eff}$ represent the overall effective active/ reactive power that supplied from (m-th) buses. Therefore, the active power losses in the lines are expressed as:


$${\text{P}}_{\text{linloss}\left(\text{rm}\right)}={\text{R}}_{\text{rm}}\frac{\left({\text{P}}_{m,eff}^{2}+{\text{jQ}}_{m,eff}^{2}\right)}{{\text{V}}_{\text{m}}^{2}}$$ (24)

Then, the LSF is utilized by obtaining first derivative of ${\text{P}}_{\text{linloss}\left(\text{rm}\right)}$ for the above equation regarding to the effective generated active power ${\text{P}}_{\text{m},\text{eff}}$ that is behind m-th buss and it is calculated as reported in [47,60].


$$\text{LSFs}=\frac{\delta {\text{P}}_{\text{linloss}\left(\text{rm}\right)}}{{\text{δP}}_{\text{m},\text{eff}}}=\frac{2{\text{*P}}_{\text{m},\text{eff}}{\text{* jR}}_{\text{rm}}}{{\text{V}}_{\text{m}}^{2}}$$ (25)

#### 2) Voltage Sensitivity Factor (VSF).

Voltage Sensitivity Factors (VSF) are parameters utilized in power system analysis to quantify the sensitivity of bus voltages to changes in system parameters, such as active power injections, reactive power injections, or network impedance. VSFs help in understanding how changes in the power system configuration or operating conditions affect the voltage shape at specific nodes. The VSF for a particular node is identified as the partial derivative of the node voltage against a particular system parameter, while keeping other parameters constant. The VSF can be performed by dividing the voltage magnitudes of base case at buses V(r) by the lower boundary of voltage (0.95 p.u.). The buses that have high records of LSF and VSF less than 1.01 are considered the most candidate nodes for RDG integration.

## III. Optimization methods

### A. Seagull Optimization Algorithm (SOA)

The mathematical model of the suggested SOA method is explained in this part. The body of most seagulls is covered by white fledge and they have different lengths and masses. These birds usually live in colonies. They eat fish, insects, eggs, reptiles, and earthworms. The search agents are intelligent birds, they have many methods to find and hunt prey such as bread crumbs which are used to attack and catch fish. Moreover, they make rain sounds with their feet to hunt invisible earthworms. The most important behavior about seagulls is migration and hunting [61,62].

#### 1) The mathematical modelling.

i) Migration (exploration)

In migration, the proposed methodology discusses the movement of seagulls from one position to another. The collisions among neighbors can be bypassed by modifying their location using a variable ${\text{M}}_{\text{a}}$.


$${\text{P}}_{\text{n}}\left(\text{y}\right)={\text{M}}_{\text{a}}\times {\text{P}}_{\text{i}}\left(\text{y}\right)$$ (26)

where ${\text{P}}_{\text{n}}\left(\text{y}\right)$ denotes the updated location of the search agents after avoiding the collision and ${\text{P}}_{\text{i}}\left(\text{y}\right)$ denotes the initial location of the agents, y defines the present iteration, and ${\text{M}}_{\text{a}}$ is the seagull’s movement which is determined using:


$${\text{M}}_{\text{a}}=\text{Z}-\left(\text{Z}\times \frac{\text{w}}{{\text{w}}_{\text{max}}}\right)$$ (27)

and w = (0, 1,…, ${\text{w}}_{\text{max}}$).

where Z decreases linearly to 0 and ${\text{M}}_{\text{a}}$ begins at Z and terminates at 0 when ${\text{w}}_{\text{max}}$ is achieved. After avoiding collisions with other birds, the agents move to the finest search space which is illustrated as below:


$${\text{P}}_{\text{t}\_\text{best}}\left(\text{y}\right)=\text{Q}\times \left({\text{P}}_{\text{best}}\left(\text{y}\right)-{\text{P}}_{\text{i}}\left(\text{y}\right)\right)$$ (28)

where ${\text{P}}_{\text{t}\_\text{best}}(\text{y)}$ is the location in the way of the finest agents, ${\text{P}}_{\text{best}}\left(\text{y}\right)$ denotes the improved place in the searching space, and Q is a vector for balancing exploration and exploitation which is calculated by:


Q=2×D×D×ran (29)

Finally, the seagulls can update their places to reach the new best location regarding the best agent.


$${\text{P}}_{\text{f}{\_}_{\text{best}}}\left(\text{y}\right)=│{\text{P}}_{\text{n}}\left(\text{y}\right)+{\text{P}}_{\text{t}\_\text{best}}\left(\text{y}\right)│$$ (30)

where ${\text{P}}_{\text{f}\_\text{best}}\left(\text{y}\right)$ is the spacing between the seagull and the best one.

ii) The Attack of seagulls (exploitation)

In attacking, the speed and angle of the seagulls can be changed in the direction of the prey in spiral 3D movement behavior as follows:


$$\text{A}=\text{q}\times \cos\left(\text{θ}\right)$$ (31)

$$\text{B}=\text{q}\times \sin\left(\text{θ}\right)$$ (32)

**Fig 2:**
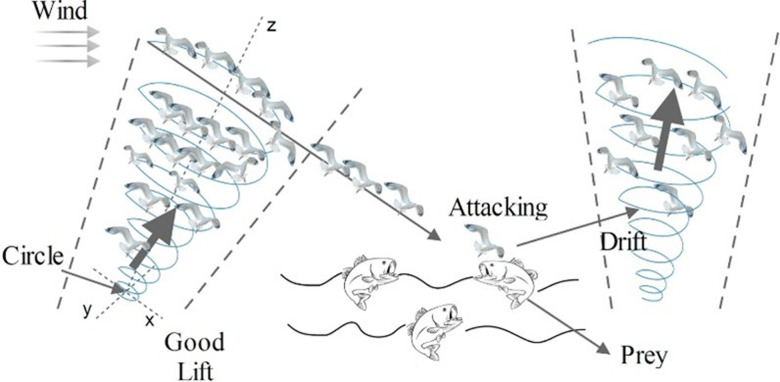
Migration and attacking prey.

**Fig 3:**
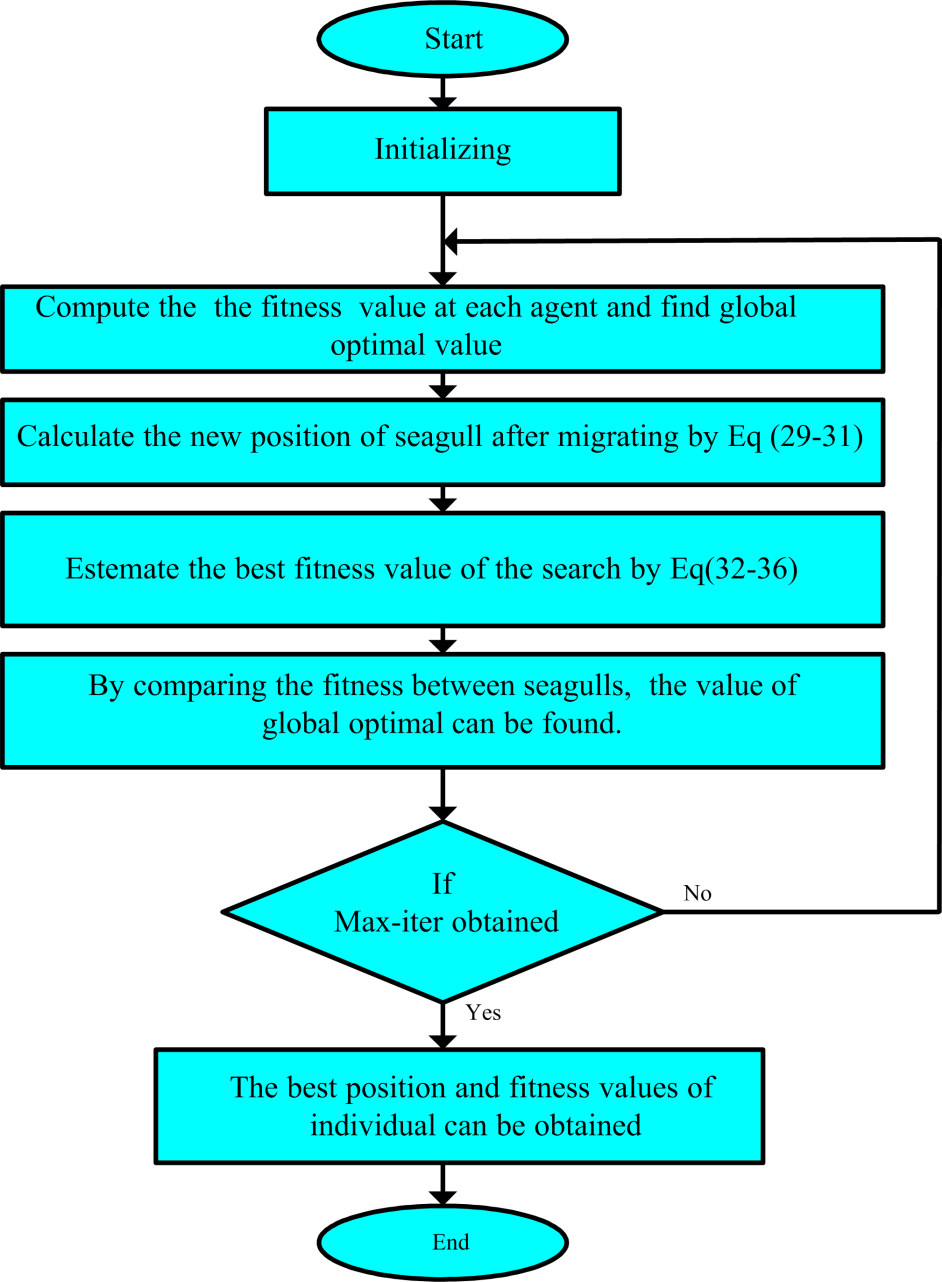
Flowchart of the suggested SOA.

C=q×θ (33)

$$\text{q}=\text{γ}\times {\text{e}}^{\text{θl}}$$ (34)

where q represents the radius of each spiral, γ and l  are constant variables defining the shape of spiral, and θ is the angle of the spiral in range of [0 ≤θ≤2π]. Equation (36) illustrates the updated position of seagulls:


$${\text{P}}_{\text{i}}\left(\text{y}\right)=\left(\text{A}\times \text{B}\times \text{C}\times {\text{P}}_{{\text{f}}_{\_\text{best}}}\left(\text{y}\right)\right)+{\text{P}}_{\text{best}}\left(\text{y}\right)$$ (35)

where ${\text{P}}_{\text{i}}\left(\text{y}\right)$ records the best result and modifies the places of other individuals. The procedure of migrating and attacking of agents can be shown in Fig 2. Then, Fig 3 describes the seagull optimization algorithm.

### B. Marine Predators Algorithm (MPA)

The MPA approach is detailed in a previous publication [63]. In this technique, both the predator and victim were considered as search agents, as the prey is actively engaged in seeking sustenance while the predator is in pursuit. This symbiotic interaction occurs as the predator searches for its victim, and concurrently, the victim endeavors to secure its nutrition [64]. The initial solution is a uniform distribution over the problem area of the problem based on the next equation:


$${\text{X}}_{0}={\text{X}}_{\text{min}}+\text{rand}\left({\text{X}}_{\text{max}}-{\text{X}}_{\text{min}}\right)$$ (36)

where ${\text{X}}_{0}$ indicates the initialed parameters, rand is an arbitrary number ranged between [0 1], ${\text{X}}_{\text{min}}$ and ${\text{X}}_{\text{max}}$ signify the upper and lower boundaries for variable ${\text{X}}_{0}$. Then, the MP fitness can be estimated by the existence of the fittest theorem [65].

According to the « survival of the fittest theory, predators have to select an optimum method to maximize their obverse rates with prey in nature. So, the finest solution is a top MP to establish a matrix elite demonstrated as follows:


$$\text{elite}=\left[\begin{array}{c} \begin{array}{cc} {\text{X}}_{1,1}^{1} & {\text{X}}_{1,2}^{1}\\ {\text{X}}_{2,1}^{1} & {\text{X}}_{2,2}^{2} \end{array}\\ \begin{array}{cc} \ldots . & \ldots .\\ {\text{X}}_{\text{n},1}^{1} & \ldots . \end{array} \end{array}\begin{array}{c} \begin{array}{cc} \ldots . & {\text{X}}_{1,\text{d}}^{1}\\ \ldots . & {\text{X}}_{2,\text{d}}^{1} \end{array}\\ \begin{array}{cc} \ldots . & \ldots .\\ \ldots . & {\text{X}}_{\text{n},\text{d}}^{1} \end{array} \end{array}\right]$$ (37)

The location of the predator can be modified by another matrix named prey. The main role of this matrix organizes the process of finding and searching the prey according to the data of prey’s positions.


$$\text{prey}=\left[\begin{array}{c} \begin{array}{cc} {\text{X}}_{1,1} & {\text{X}}_{1,2}\\ {\text{X}}_{2,1} & {\text{X}}_{2,2} \end{array}\\ \begin{array}{cc} \ldots . & \ldots .\\ {\text{X}}_{\text{n},1} & \ldots . \end{array} \end{array}\begin{array}{c} \begin{array}{cc} \ldots . & {\text{X}}_{1,\text{d}}\\ \ldots . & {\text{X}}_{2,\text{d}} \end{array}\\ \begin{array}{cc} \ldots . & \ldots .\\ \ldots . & {\text{X}}_{\text{n},\text{d}} \end{array} \end{array}\right]$$ (38)

The MPA methodology is categorized into three phases in accordance with the different velocity rates.

#### 1) Stage 1: High velocity ratio.

It takes place when prey is running quicker than a predator. This scheme occurs in the premier iterations of the optimization procedure, $\text{itr}<\frac{1}{3}{\text{max}}_{\text{i}}$, in high velocity ratio (V ≥ 10) which is associated with high exploration capability.


$${\text{stepsize}}_{\text{j}}={\text{U}}_{\text{B}}⊗\left({\text{elite}}_{\text{j}}-{\text{K}}_{\text{B}}{\text{Prey}}_{\text{j}}\right)\text{j}=1,\ldots ...\ldots ,\text{n}$$ (39)

$${\text{Prey}}_{\text{j}}={\text{Prey}}_{\text{j}}+\text{J}.\text{U}⊗{\text{stepsize}}_{\text{j}}$$ (40)

where itr denotes the current iteration, and ${\text{max}}_{\text{i}}$ signifies the max iterations, ${\text{U}}_{\text{B}}$ and U are randomly chosen numbers. The first element simulates the movement of prey and the second is a vector between zero and one. ⨂  denotes the entry wise multiplications. J signifies a constant parameter.

#### 2) Stage 2: Unit velocity ratio (V ~1).

It takes place when predator and victim are running with equal velocity. Gradually, the exploration is changed to exploitation in the middle stage of optimization procedure; in this stage predator and prey are looking for their nutrition sources.


$$\frac{1}{3}{\text{max}}_{\text{i}tr}<itr<\frac{2}{3}{\text{max}}_{\text{i}tr}$$

The population is separated into two sections: Half of the iterations are assigned to exploration and the other part is assigned to exploitation.

#### 3) The first half of the iterations.

In this part, the prey’s motion is quicker than predator; therefore, the predator is responsible for exploration and prey for exploitation.


$${\text{stepsize}}_{\text{j}}={\text{U}}_{\text{L}}⊗\left({\text{elite}}_{\text{j}}-{\text{U}}_{\text{L}}{\text{Prey}}_{\text{j}}\right)\text{j}=1,\ldots \ldots ,\text{n}/2$$ (41)

$${\text{stepsize}}_{\text{j}}={\text{U}}_{\text{B}}⊗\left({\text{U}}_{\text{B}}⊗{\text{elite}}_{\text{j}}-{\text{Prey}}_{\text{j}}\right)\text{j}=1,\ldots \ldots ,\text{n}/2,\text{n}$$ (42)

where ${\text{U}}_{\text{L}}$ is the behavior of Levy flight.

#### 4) The other part of the iterations.

The prey’s position can be updated according to the movement of predators in Brownian motion.


$${\text{stepsize}}_{\text{j}}={\text{U}}_{\text{B}}⊗\left({\text{U}}_{\text{B}}⊗{\text{elite}}_{\text{j}}-{\text{Prey}}_{\text{j}}\right)\text{j}=1,\ldots \ldots ,\text{n}$$ (43)

$${\text{Prey}}_{\text{j}}={\text{Elite}}_{\text{j}}+\text{J}.\text{CF}⊗{\text{stepsize}}_{\text{j}}$$ (44)

where ${\text{A}}_{\text{p}}$ denotes to an adaptive parameter employed for adjusting the movement size of the predator and can be determined by:


$${\text{A}}_{\text{p}}={\left(1-\frac{\text{itr}}{\text{max}\_\text{itr}}\right)}^{2\frac{\text{itr}}{\text{max}\_\text{itr}}}$$ (45)

#### 5) Stage 3: Low-velocity ratio $\left(V=0.1\right)$ .

**Fig 4:**
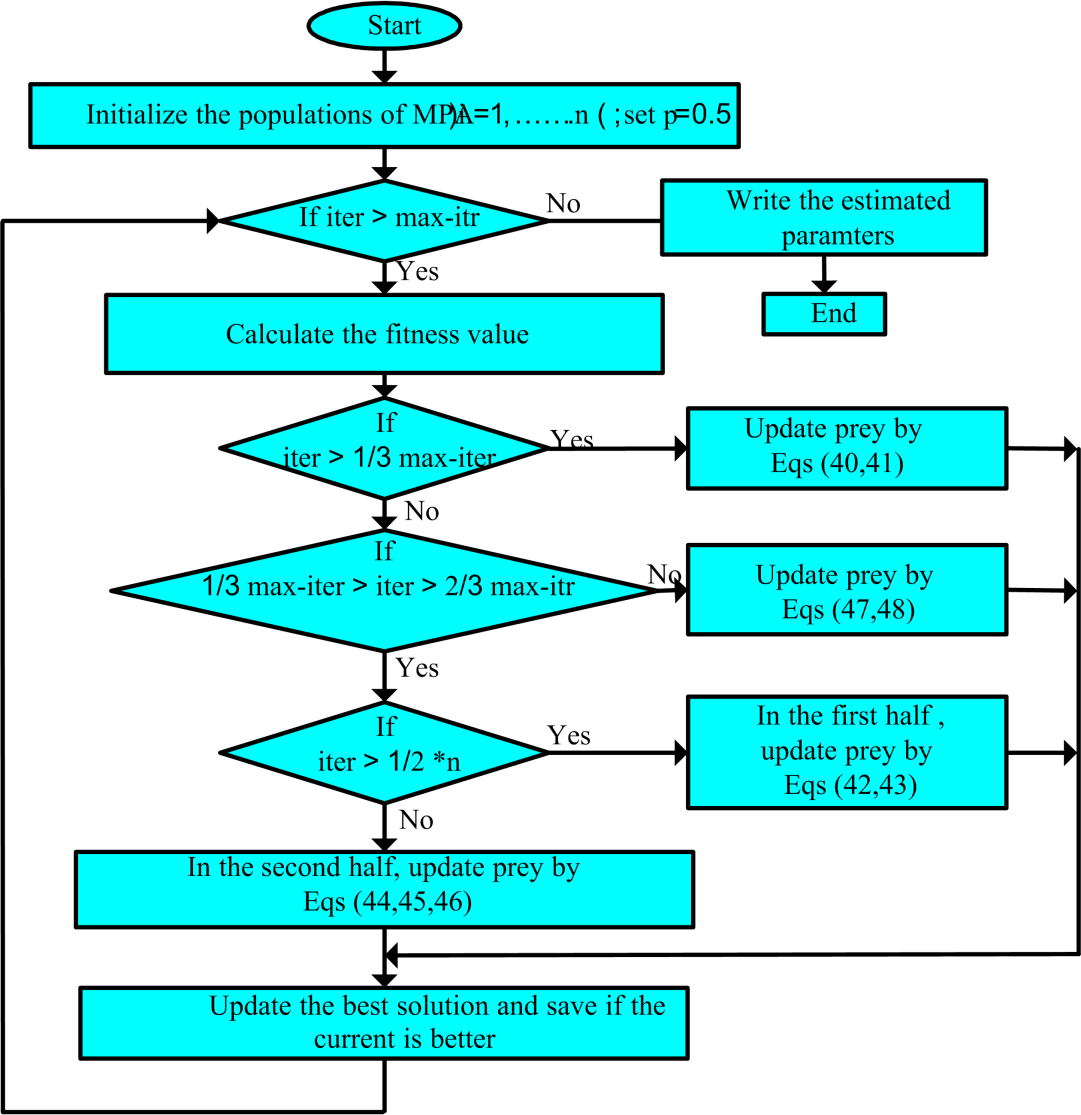
Flowchart of marine predator algorithm.


It takes place when the predator is running faster than its victim. This section takes place in the last section of the MPA methodology for high exploiting ability. Fig 4 illustrates the flowchart of MPA.

$$\text{itr}<\frac{2}{3}{\text{max}}_{\text{itr}}$$

$${\text{stepsize}}_{\text{j}}={\text{U}}_{\text{L}}⊗\left({\text{U}}_{\text{L}}⊗{\text{Elite}}_{\text{j}}-{\text{Prey}}_{\text{j}}\right)\text{j}=1,\ldots \ldots ,\text{n}$$ (46)

$${\text{Prey}}_{\text{j}}={\text{Elite}}_{\text{j}}+\text{J}.{\text{A}}_{\text{p}}⊗{\text{stepsize}}_{\text{j}}$$ (47)

### C. Salp Swarm Algorithm (SSA)

#### 1) Inspiration of SSA.

**Fig 5:**
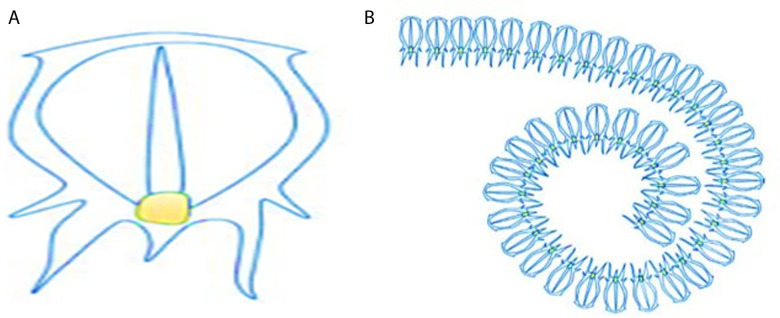
(a) illustrates the single salp. (b) Swarm of salps.

The primary source of inspiration for the salp algorithm stems from the swarming behavior exhibited by salps as they navigate and search for sources of nutrition in oceanic environments. Salps, belonging to the salpidae family, share similarities in movement and tissue structure with jellyfish. This unique biological design enables salps to expel water from their gelatinous bodies through internal feeding filters [66]. A schematic of individual salp is described in Fig 5(a). In oceans, salps are grouped with each other to form a salp chain. The salp chain is shown in Fig 5(b).

In modeling, the swarm of salps is characterized into two groups, leader and the rest of slaps. The leader is the primacy of the group and guides the salps for finding the food and hunting the prey while the followers follow their leader [66].The search area of n-dimensional is observed as the location of swarms, where Z refers to the location of a salp, n signifies variable number for each problem. Therefore, the places of salps in the swarm are kept in the matrix Z, expressed in Eq. (48). ${\text{K}}_{\text{m}}$ refers to the source of food in search space.


$$Z_{\text{m}}=\left[\begin{array}{c} \begin{array}{cc} {\text{z}}_{1}^{1} & {\text{z}}_{2}^{1}\\ {\text{z}}_{1}^{2} & {\text{z}}_{2}^{2} \end{array}\\ \begin{array}{cc} \ldots . & \ldots .\\ {\text{z}}_{1}^{\text{n}} & {\text{z}}_{2}^{\text{n}} \end{array} \end{array}\begin{array}{c} \begin{array}{cc} \ldots . & {\text{z}}_{\text{d}}^{1}\\ \ldots . & {\text{z}}_{\text{d}}^{2} \end{array}\\ \begin{array}{cc} \ldots . & \ldots .\\ \ldots . & {\text{z}}_{\text{d}}^{\text{n}} \end{array} \end{array}\right]$$ (48)

The leader’s location is modified by the next formula:


$${\text{Z}}_{\text{j}}^{1}=\left\{\begin{array}{c} {\text{K}}_{\text{m}}+{\text{d}}_{1}(\left({\text{S}}_{\text{bm}}-{\text{G}}_{\text{bm}}\right){\text{d}}_{2}+{\text{G}}_{\text{bm}},{\text{d}}_{3}\geq 0\\ {\text{K}}_{\text{m}}-{\text{d}}_{1}(\left({\text{S}}_{\text{bm}}-{\text{G}}_{\text{bm}}\right){\text{d}}_{2}+{\text{G}}_{\text{bm}},{\text{d}}_{3}<0 \end{array}\right\}$$ (49)

where ${\text{Z}}_{\text{m}}^{1}$ denotes the first positions of salp and ${\text{K}}_{\text{m}}$ indicates the place of nutrition supply in m-th dimension, ${\text{S}}_{\text{bm}}$ and ${\text{G}}_{\text{bm}}$ represents the higher and lower boundaries in m-th dimension, where ${\text{d}}_{1},{\text{d}}_{2},$ and ${\text{d}}_{3}$ signify arbitrary numbers ranged between 0 and 1. Parameter ${\text{d}}_{1}$ is the most important parameter in SSA due to its ability in balancing between the exploration and exploitation stages and is represented as:


$${\text{d}}_{1}=2{\text{e}}^{-{\left(\frac{4l}{\text{L}}\right)}^{2}}$$ (50)

where L denotes to the maximum iterations and l indicates the present iteration. The locations of the followers are modified according to newton’s law of motion by the following equation.


$${\text{z}}_{\text{m}}^{\text{i}}=\frac{1}{2}{\text{bt}}^{2}+{\text{tv}}_{0}$$ (51)

where i≥2, and ${\text{z}}_{\text{m}}^{\text{i}}$ is the position of the rest of salps and ${\text{v}}_{0}$ represents the initial of speed.


$$\text{b}=\frac{{\text{v}}_{\text{final}}}{{\text{v}}_{0}},\text{and v}=\frac{\text{z}-{\text{z}}_{0}}{\text{t}}$$ (52)

During optimization models, time might be treated as an iteration. Therefore, the step change between iterations equals to 1, and it is assumed that ${\text{v}}_{0}=0$, so,


$${\text{z}}_{\text{m}}^{\text{i}}=\frac{1}{2}\left({\text{z}}_{\text{m}}^{\text{i}}+{\text{z}}_{\text{m}}^{\text{i}-1}\right)$$ (53)

**Fig 6:**
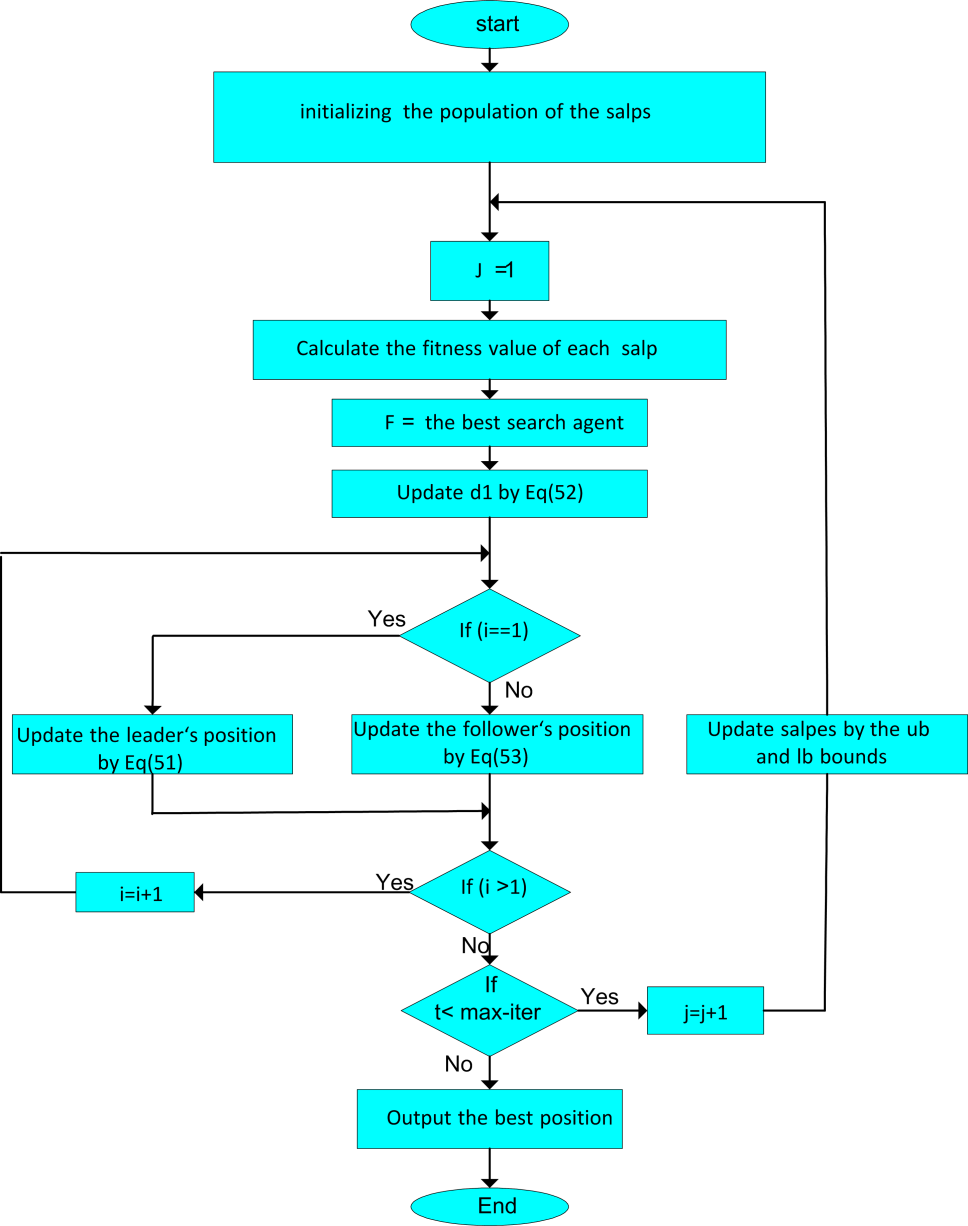
Flowchart of the suggested SSA methodology.

For finding the global optimum, the SSA begins the optimization process with many individuals with random places. After that, the places of leader and his followers will be updated recursively by Eqs. (50) and (53), and eventually, the global optimum solution of SSA will be found. The process of the SSA methodology is illustrated in Fig 6.

#### 2) Exploration and exploitation.

The overarching goal of exploration within optimization algorithms is to unveil and traverse the search spaces effectively. This exploration process aids the solver in navigating the vast solution space, enhancing the likelihood of identifying the optimum location and maximizing the chance of discovering the most favorable minima. Essentially, exploration is about broadening the search scope, allowing the algorithm to venture into diverse regions of the solution space [67]. Conversely, exploitation is focused on refining the search by homing in on the most promising solutions within the identified regions. The objective is to attain the best possible solutions by strategically locating the finest solution candidates. Exploitation involves a more in-depth analysis of specific areas within the solution space that exhibits potential for optimal outcomes. Balancing exploration and exploitation is a critical aspect of optimization algorithms, as it ensures a comprehensive search for the global optimum while also refining the search in promising regions to achieve high-quality solutions

In SSA, coefficient ${\text{d}}_{1}$ is incorporated for balancing between exploration and exploitation phases and is presented in Eq. (50). The downside of SSA is the tardy convergence speed [68].

### D. Grey Wolf Optimizer (GWO)

GWO methodology is discussed in [69]. Grey wolves are categorized in four main categories: alpha, beta, delta, and omega for representing the leadership hierarchy. Then, the steps of hunting are employed such as searching, surrounding, and attacking prey.

#### 1) Searching prey.

In the grey wolf algorithm, most grey wolves prefer to live in a group of an average 5 - 12 individuals. The α wolf is the leader of the herd and is accountable for taking decisions about hunting, feeding and sleeping place and its order is obeyed by the rest of the group. The second type in the herd is known as β that assist the leader for attacking the prey and other pack activities, the third is called delta and ω wolf stands for the rest of the herd. Their behavior is high-flown by the first three categories and comply with their dictations. Wolves anticipate this hierarchy for foraging and attacking the victim. α, β, and δ wolves are nearby to capture the prey, and ω wolves follow the first three wolves to search, prosecute, and hunt their prey. When the surrounding circle is quite small, the wolves start to attack and chase their victim.

#### 2) Encircling prey.

In the algorithm, the location of agents is updated based on equations (54) and (55):


$$\vec{Q}=|\text{W}.\vec{Y}\text{(t) -}{\vec{Y}}_{p}\text{(t) |}$$ (54)

$$\vec{D}(t+1)=\vec{Y}\text{(t) }-C.\vec{Q}$$ (55)

Here Q signifies the spacing between the wolf and the defined victim, t signifies the current iteration, $\vec{\text{C }}$ and $\vec{\text{W }}$ signify the coefficient vectors, ${\text{x}}_{\text{p}}$ signifies the place of prey, and $\vec{\text{D }}$ implies a vector describing the location of the wolf. $\vec{\text{C }}$ and $\vec{\text{W }}$ are evaluated using equations 57 and 58:


$$\vec{C}=2\text{r}.{\vec{j}}_{1}-\vec{r}$$ (56)

$$\vec{W}=2.{\vec{j}}_{2}$$ (57)

where $\vec{r}$ is the coefficient that reduced from 2 to 0 with going on the optimization process and the values of ${\text{j}}_{1}$ and ${\text{j}}_{2}$ are randomly located between 0 and 1.

#### 3) Attacking prey.

**Fig 7:**
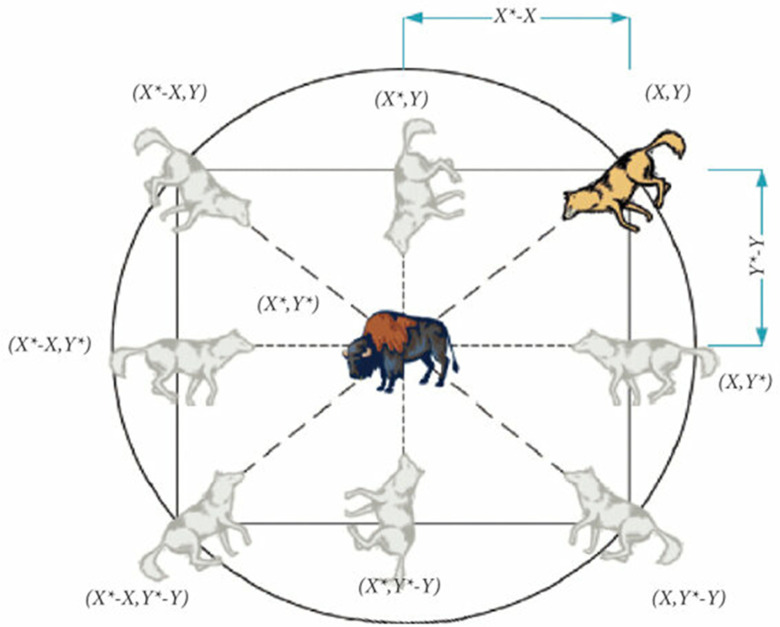
Position vectors and their possible next locations.


As shown in Fig 7, the grey wolf firstly identifies the place of the victim and surrounds them for hunting. The hunting procedure is controlled by alpha (α), beta (β), and delta (δ). Therefore, alpha is considered the finest solution obtained, (β) and (δ) are treated as the finest candidates. However, the other search agents (named omega wolves) should relocate their places following the best solution alpha, beta, and delta which were recorded before [70]. The modelling of hunting performance of the search agent is presented in (58).

$$|{\vec{Q}}_{\text{α}}={\text{W}}_{1}.{\text{Y}}_{\text{α}}–\text{Y}│$$

$$|{\vec{Q}}_{\text{β}}={\text{W}}_{2}.{\text{Y}}_{\text{β}}–\text{Y| }$$

$$|{\vec{Q}}_{\delta }={\text{W}}_{3}.Y_{\text{δ}}–\text{Y| }$$ (58)

where ${\text{Y}}_{\text{α}}$ is the optimal solution, ${\text{Y}}_{\text{β}}$ implies the second solution, and ${\text{Y}}_{\text{δ}}$ is the third solution in a given iteration t.

The present location of ω wolves regarding each best solution is shown below:


$${\vec{Y}}_{1}={\vec{Y}}_{a}–{\text{E}}_{1}.({\vec{Q}}_{a})$$

$${\vec{Y}}_{2}={\vec{Y}}_{\beta }–{\text{E}}_{2}.({\vec{Q}}_{\beta })$$

$${\vec{Y}}_{3}={\vec{Y}}_{\delta }–E_{3}.({\vec{Q}}_{\delta })$$ (59)

$$\vec{Y}(t+1)=\frac{{\vec{\text{Y }}}_{1}+{\vec{\text{Y }}}_{2}+{\vec{\text{Y }}}_{3}}{3}$$ (60)

**Fig 8:**
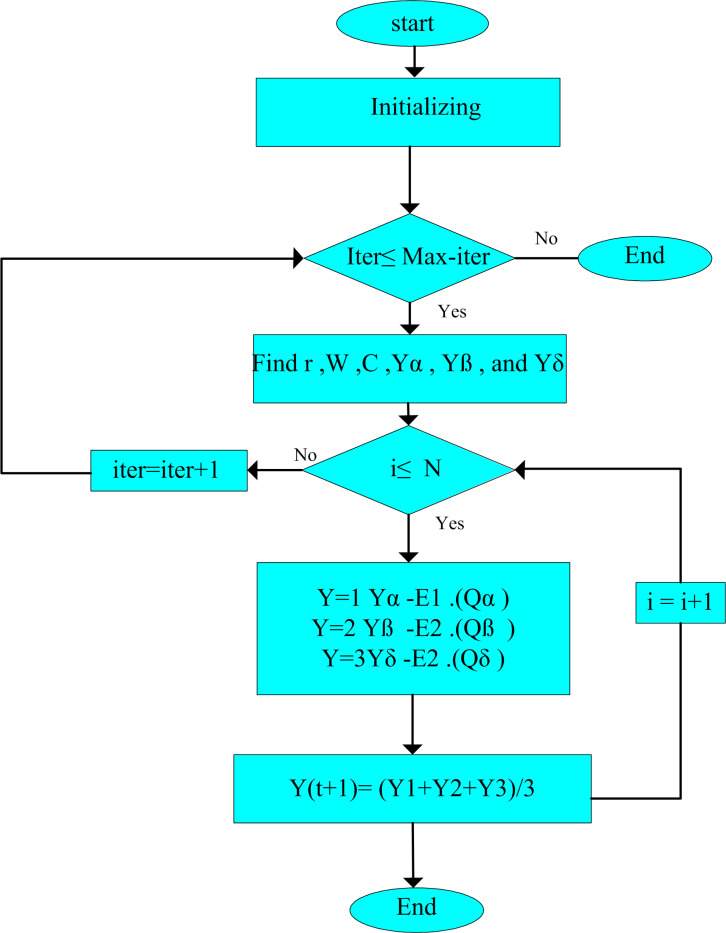
Description of the behaviour of GWO.

Fig 7 illustrates Eq. (60), describing the way that a search agent modifies its location, following alpha, beta, and delta in the problem area. The grey wolves can modify their location surrounding the prey after finding the prey’s location. Fig 8 illustrates the flowchart of the suggested GWO.

### E. Improved grey wolf optimizer

**Fig 9:**
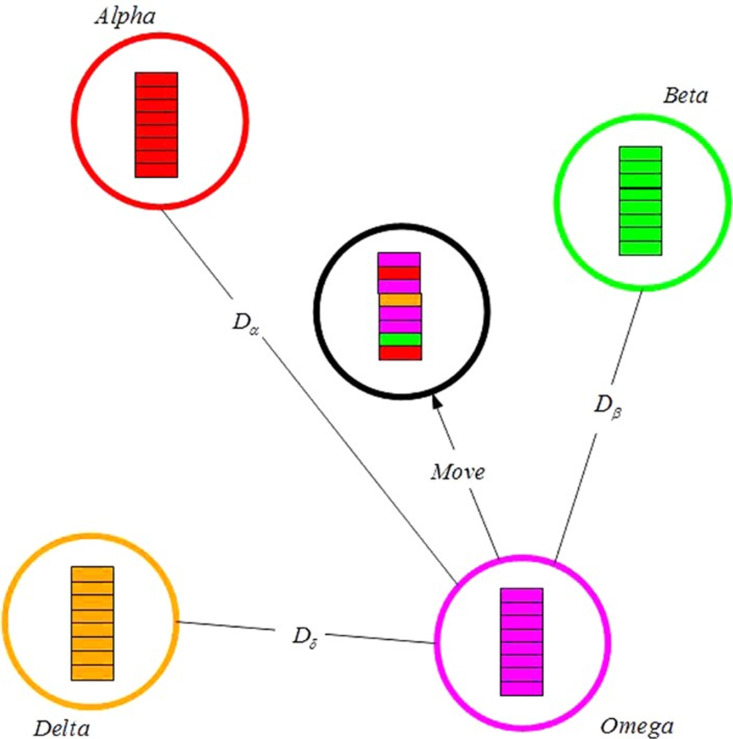
The updating positions of IGWO.

The IGWO was provided to overcome the disadvantages of GWO such as the local minima and slow convergence rate. These issues are avoided by updating the mechanism of GWO, which focuses on an appropriate balancing between exploration and exploitation. The wolf’s location is modified based on the “survival of the fittest” theorem to avoid from getting staked into the local optimum [71]. At the end of every iteration, the IGWO methodology saves the fitness values for each wolf by ascending order. After that, R wolves having less fitness are outcasted, and fresh R wolves are arbitrarily produced instead of the eliminated wolves. IGWO consists of three main steps: initializing, movement, and updating. The location of the wolves is changed, as described in Fig 9. The improvements contain a new search strategy related to the updating stage [72].

#### 1) Initializing.

In this step, R wolves are randomly spread in the search area in two individuals of parent ($Y_{\text{b}1},Y_{\text{b}2}$).


$${\text{Y}}_{\text{i}}\left(\text{t}+1\right)=Y_{\text{b}3}+{\text{s}}_{\text{f}}\text{*}\left(Y_{\text{b}1}-Y_{\text{b}2}\right)$$ (61)

Here ${\text{b}}_{1}$ and ${\text{b}}_{2}$ refer to the index numbers of the parents of the search agents. After a number of simulations, α wolf is selected to form a variation factor and beta and delta are treated as two parents, as illustrated in Eq. (62). Thus, the initializing is shown as follows:


$${\text{Y}}_{\text{i}}\left(\text{t}+1\right)=Y_{\text{α}}+{\text{s}}_{\text{f}}\text{*}\left(Y_{\text{β}}-Y_{\text{δ}}\right)$$ (62)

where ${\text{b}}_{1}$, ${\text{b}}_{2}$ and ${\text{b}}_{3}$ represent the various integers in (1, 2, … n), and ${\text{s}}_{\text{f}}$ is a scaling parameter which is utilized to enhance the system’s convergence rate and bypassing the local minima. So, the scaling coefficient ${\text{s}}_{\text{f}}$ is sorted from small to large with advance in the simulation as in Eq. (63).


$${\text{s}}_{\text{f}}={\text{s}}_{\text{min}}+\left({\text{s}}_{\text{f min}}-{\text{s}}_{\text{f max}}\right)\text{*}\frac{{\text{max}}_{\text{iter}}-\left(\text{iter}-1\right)}{{\text{max}}_{\text{iter}}}$$ (63)

where ${\text{s}}_{\text{fmax}}{\text{and s}}_{\text{fmin}}$ denote the max and min values of the scaling factor, and iter is current iteration.

#### 2) Movement stage.

In the standard GWO, a new position is produced for every wolf with the help of three leader wolves (α,β and δ) to achieve the optimal solution. This mechanism may lead GWO getting staked in the local optimum and showing low convergence rate. To conquer these drawbacks and improve the GWO, an additional moving methodology called dimension learning-based hunting (DLH) approach is proposed. In the DLH search strategy, each single wolf is known by its different neighbors and a randomly selected wolf from Population is calculated by Eq. (64). Then, another candidate for the modified location of ${\text{Y}}_{\text{i}}\left(\text{t}\right)$ named ${\text{Y}}_{\text{i}-\text{DLH}}\left(t+1\right)$ is generated by DLH search strategy. To do this, at the beginning, a radius ${\text{R}}_{\text{i}}\left(\text{t}\right)$ is evaluated using Euclidean distance between the present location of ${\text{Y}}_{\text{i}}\left(\text{t}\right)$ and the candidate position ${\text{Y}}_{\text{i}-\text{GWO}}\left(\text{t}+1\right)$ according to 65.


$${\text{R}}_{\text{i}}\left(\text{t}\right)=|{\text{Y}}_{\text{i}}\left(\text{t}\right)-{\text{Y}}_{\text{i}-\text{GWO}}\left(\text{t}+1\right)|$$ (64)

$${\text{Y}}_{\text{i}-\text{DLH}}\left(t+1\right)={\text{Y}}_{\text{i},d}\left(\text{t}\right)+rand\text{* }({\text{Y}}_{\text{n},d}\left(\text{t}\right)-{\text{Y}}_{\text{r},d}\left(\text{t}\right)$$ (65)

#### 3) Updating stage.

During this stage, firstly, the fitness of two individuals $Y_{\text{i}-\text{DLH}}\left(t+1\right)$ and ${\text{Y}}_{\text{i}-\text{GWO}}\left(t+1\right)$ are compared to select the superior candidate by the following equation:


$${\text{Y}}_{\text{i}}\left(\text{t}+1\right)=\left\{\begin{array}{c} {\text{Y}}_{\text{i}-\text{GWO}}\left(\text{t}+1\right),iff\left({\text{Y}}_{\text{i}-\text{GWO}}\right)<{\text{Y}}_{\text{i}-\text{DLH}}\\ {\text{Y}}_{\text{i}-\text{DLH}}\left(\text{t}+1\right)otherwise \end{array}\right\}$$ (66)

**Fig 10:**
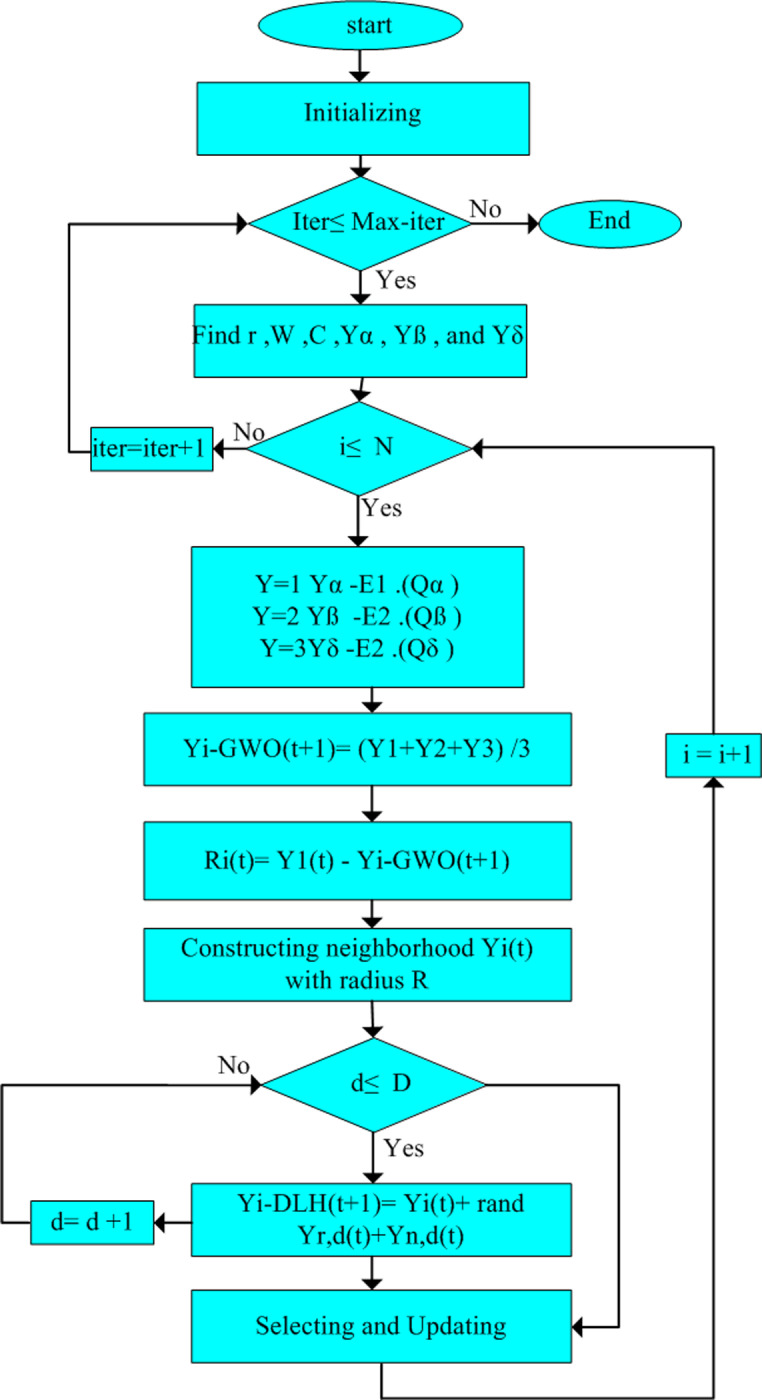
Flowchart of I-GWO.

The fitness for each individual is sorted from the smallest to the largest. Then, the updated location of ${\text{Y}}_{\text{i}}\left(\text{t}+1\right)$ is updated, when the fitness of the chosen candidate is lower than ${\text{Y}}_{\text{i}}\left(\text{t}\right)$. Otherwise, ${\text{Y}}_{\text{i}}\left(\text{t}\right)$ remains unchanged in the population. In the first half of iterations, the population’s difference is large, so the modified methodology has robust exploration capability. But, in the last half of simulation, when the IGWO converges, difference between wolves is insignificant, so the technique has durable exploitation characteristic [72]. The suggested I-GWO methodology is presented in Fig 10.

### F. Application of optimization methods for DG allocation

Various optimization algorithms of SSA, MPA, GWO, IGWO, and SOA are applied for the optimal placement of Distributed Generation (DG) within a distribution system. A general procedure following these steps can be applied for the application of each algorithm individually:

Step 1.Input Data and Objective Definition, which include the line data and load data, is input, and the objective function is formulated to represent the optimization goal.
Step 2.Algorithm initialization in which the population, algorithm-specific parameters, and the maximum number of iterations (or stopping criteria) are initialized.
Step 3.Objective Function Evaluation, in which the power flow analysis is performed, and the objective function is evaluated for each individual or candidate solution in the population considering E1. (18).
Step 4.The positions or values of the candidate solutions are updated according to the unique rules and mechanisms defined by the selected algorithm. It is important to note that these rules and mechanisms vary significantly across different algorithms. For instance, the update rules in SOA are mathematically defined in Eqs. (28–30), while Eqs. (50) and (52) govern the update process in SSA.
Step 5.Diversity preservation and selection mechanisms (e.g., mutation, crossover, or leader-based selection) are applied, as defined by the algorithm.
Step 6.Power flow analysis is repeated, the objective function is recalculated, and the best solution is updated and stored.
Step 7.Check If the current iteration or stopping condition is not satisfied, the process loops back to Step 4. Otherwise, proceed to the next step.
Step 8.The final Optimal solution, including DG sizes and their optimal locations, is returned as the output.

The presented approach steps ensure flexibility in applying the tested different optimization methods while maintaining a structured framework for efficient DG allocation, enhancing the performance and reliability of the distribution system.

## IV. Simulation results

In this study, the performance of the SSA, MPA, GWO, IGWO, and SOA has been assessed using IEEE 33-bus and 69-bus Radial Distribution Systems (RDS) operating at 12.66 kV [47]. The voltage magnitude bounds for the systems under investigation are set at 1.05 and 0.95 per unit (p.u.). To guide the multi-objective optimization process, weighting factors λ1  =  0.5, λ2  =  0.4, and λ3  =  0.1 are employed [47]. The cost coefficients, κ1  =  4 $/kW and κ2  =  5 $/kW, are selected based on existing literature [47], with κ2 representing the cost associated with the installation and maintenance of renewable distributed generations. A BFS load flow analysis is conducted to determine the voltage profile and reduce power losses in the distribution network. Sensitivity factors are incorporated to identify candidate buses suitable for integrating Photovoltaic systems into the radial distribution network. Specifically, buses with Voltage Sensitivity Factors below 1.01 p.u. are designated as candidate nodes for RDGs integration. The suggested methods are then applied to ascertain the optimal place and size of RDGs integrated into the proposed candidate nodes. In this context, the distributed generation is defined as a PV system operating at unity power factor, capable of supplying real power to the distribution network. Table 1 provides the control parameters for the suggested optimization algorithms, along with the upper and lower operating boundaries.

**Table 1:** The operating boundaries and control variables of the suggested algorithms.

Parameters	Used Value
Maximum iteration	100
Search agent number	70
V_max_, V_min_	[0,95–1.05]
[P_DG_min_, P_DG_max_]	[0–2229]

There are two cases are employed in the two test systems (33-bus and 69-bus IEEE systems):

Case 1: Without DG (Base case).
Case 2: With integrating DG.

Additionally, a comprehensive comparison between the suggested methodology and other methodologies has been performed.

### A. IEEE 33-bus system

**Fig 11:**
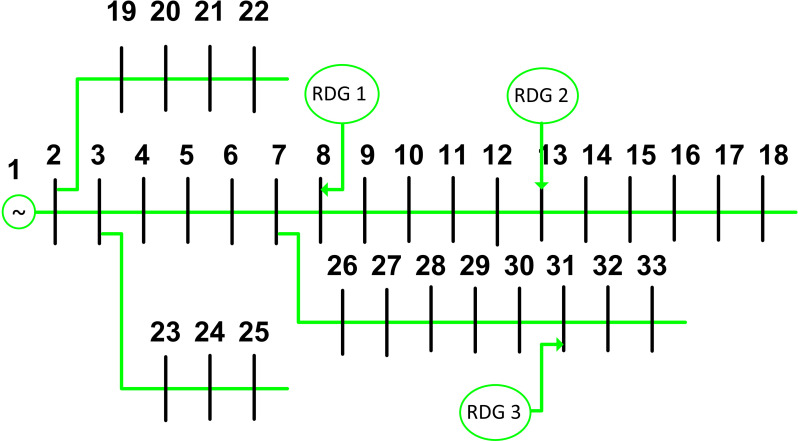
IEE 33-bus system RDG units at optimal locations.

The devised approaches have undergone testing on the IEEE 33-bus system [47]. In this context, the base voltage is standardized at 12.66 kV, while the base MVA is set at 100 MVA. For the optimization process, three units of renewable distributed generations are considered. The optimal placing of these three RDGs within the IEEE 33-bus system is visually represented in Fig 11.

Prior to the introduction of distributed generations into the radial distribution grid, the overall active power loss is documented at 210.997 kW. The lowest value of voltage amplitude is recorded as 0.9038 per unit (p.u.) and observed at node 18. To pinpoint the suitable buses for RDG installations, the VSF and LSF are analyzed for the IEEE 33-bus network. Examining Table 2 reveals the lowest VSF values coupled with the highest LSF values, aiding in the identification of appropriate buses for RDG installations.

**Table 2:** The values of LSF and VSF of the standard IEEE 33-bus.

33-Bus	LSF	VSF
6	0.02330	0.99946
8	0.02150	0.98137
28	0.01213	0.98268
9	0.01012	0.97471
13	0.00992	0.95952
10	0.00946	0.96853
29	0.00867	0.97403
31	0.00607	0.96590
30	0.00462	0.97029
27	0.00342	0.99473
14	0.00319	0.95710
17	0.00295	0.95199
12	0.00283	0.96602
7	0.00281	0.99575
26	0.00260	0.99743
15	0.00242	0.95560
16	0.00238	0.95415
11	0.00161	0.96761
32	0.00124	0.96494
18	0.00101	0.95135
33	0.00030	0.96464

Subsequently, the proposed optimization algorithm, along with other comparison methods, is applied to the first 10 rows of candidate buses. The calculated results for the proposed optimization techniques in the IEEE 33-bus system are presented in Table 3. Notably, candidate buses with substantial effects on the distribution network when integrated with RDGs have been marked as buses 8, 13, and 31. These findings underscore the efficacy of the proposed methodologies in determining optimal RDG placement for enhancing the performance of the distribution network.

**Table 3:** Results from all techniques for pv system integration (at unity p.f) in ieee 33- bus.

Results of 33- bus with PV system						
Items	Before optimization	After optimization PV system (at unity PF)				
		GWO	IGWO	SOA	SSA	MPA
Total losses (kw)	210.98	84.7976	84.7863	85.746	84.7475	84.7803
Loss reduction%	–	59.8001%	59.82%	59.36%	59.83%	59.82%
V worst (Pu), bus	0.9038, (18)	0.9635, (33)	0.9636, (18,33)	0.9606, (18)	0.9637, (18,33)	0.9636, (18,33)
Optimal location and size of DGs (kw)	–	(8)473.641 (13) 628.502 (31)746.03	(8)470.18 (13) 628.38 (31) 749.88	(8) 450 (13) 580.64 (31) 750	(8)467.84 (13) 631.09 (31) 752.77	(8) 470.46 (13)628.41 (31) 750
SDG (KVA)		1848.166	1848.448	1780.635	1851.558	1848.411
RDGs Power Factor	–	Unity	unity	unity	unity	unity
TOC ($)	–	9580	9581.4	9246	9597.1	9583.5
∆OC		0.8596	0.8587	0.8296	0.8611	0.8599
∆PlDG		0.4184	0.4184	0.4231	0.4182	0.4184
∆VD	0.0962	0.0365	0.0364	0.0394	0.0363	0.0364

The optimal capacities for the proposed PV systems at the identified buses are as follows: 467.84 kW at bus 8, 631.09 kW at bus 13, and 752.77 kW at bus 31, resulting in a total apparent power of 1851.558 kVA at unity power factor. In contrast to various techniques such as GWO, IGWO, SOA, and MPA, the SSA method yields the minimum total power losses, reducing them from 210.98 kW to 84.7475 kW, surpassing the performance of other approaches. Table 3 illustrates that the SSA algorithm not only achieves the lowest total power losses but also attains an improved minimum voltage magnitude compared to other methods. The voltage magnitude increases from 0.9037 p.u. (recorded at bus 33) to 0.9637 p.u., observed at buses 18 and 33. The reduction in total power loss with the SSA algorithm is notable, reaching 120.2325 kW, corresponding to a 59.83% decrease. Additionally, the installation of three RDG units leads to an enhanced voltage profile. Following the SOA methodology, power losses decrease to 85.746 kW, with a total ownership cost (TOC) of $9246. Meanwhile, the MPA approach reduces power losses to 84.7803 kW, achieving a TOC of $9583.5. Notably, the SSA algorithm produces a larger RDG size compared to other techniques, emphasizing its effectiveness in optimization.

**Fig 12:**
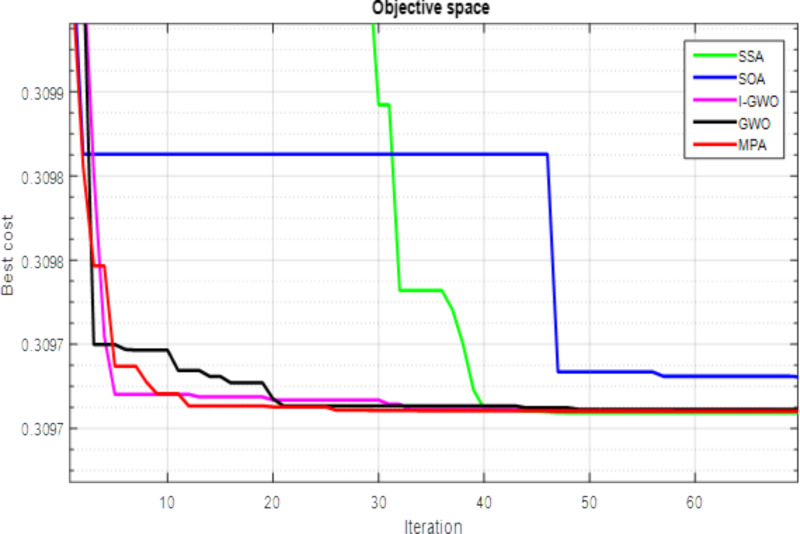
Convergence trends for IEEE 33-bus test system.

A comparative analysis of the convergence trends among the various algorithms indicates the remarkable efficiency of the Salp Swarm Algorithm in reaching the optimal solution with significantly fewer iterations, as depicted in Fig 12. The outcomes showcased in the figure distinctly demonstrate the SSA algorithm’s superior performance in comparison to other optimization techniques when addressing the optimal allocation problem associated with PV systems in the IEEE 33-bus system. The depicted convergence trends provide compelling evidence of SSA’s effectiveness in achieving convergence rapidly and attaining the optimal solution with notable computational efficiency. This establishes SSA as a robust and highly competitive optimization algorithm for addressing the complexities inherent in optimizing the allocation of PV systems within radial distribution networks, showcasing its potential for real-world applications in enhancing the efficiency of power distribution systems.

**Fig 13:**
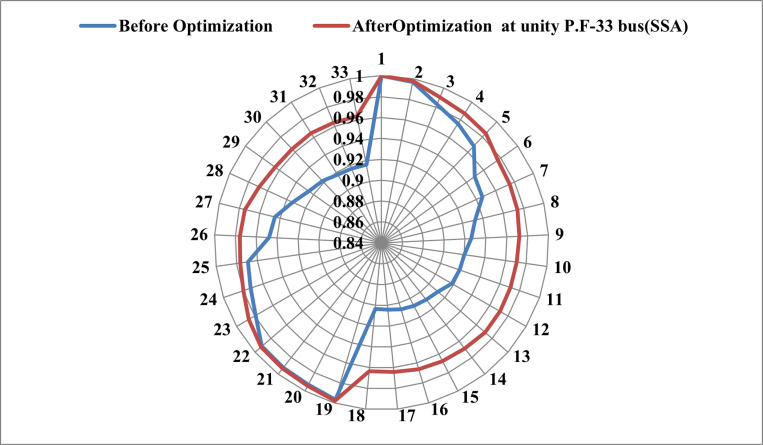
Voltage profile after integrating RDG at unity p.f. in 33-bus system using the results of SSA.

**Fig 14:**
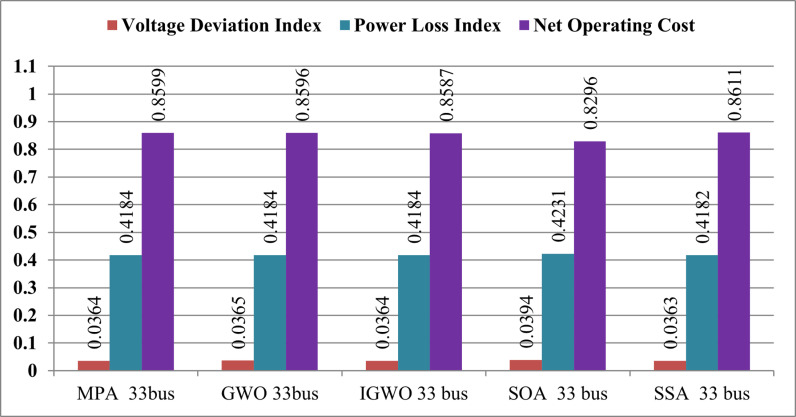
Comparison of various operation indexes for IEE 33-bus system based on all algorithms.

The radar plot portrayed in Fig 13 provides a detailed insight into the substantial improvement in the voltage shape achieved through the strategic integration of RDGs at optimal locations within the IEEE 33-bus system. Noteworthy is the discernible impact of the SSA, surpassing the performance of other algorithms and leading to a more pronounced enhancement in the voltage profile. This underscores the efficacy of SSA in achieving optimal RDG allocation and sizing, resulting in noteworthy reductions in both total operating cost and power losses. In Fig 14, a comparative analysis is presented, illustrating the performance of all techniques in relation to salp swarm algorithm. The indices for voltage deviation (∆VD) and power loss demonstrate substantial reductions when SSA is employed, outperforming alternative methods such as MPA, GWO, IGWO, and SOA. It is important to note, however, that the operating cost deviation (∆OC) with SSA is relatively higher compared to other algorithms. In summary, the results unequivocally affirm that salp swarm algorithm emerges as the most suitable methodology for achieving the highest drop in power losses and enhancing the voltage profile in the network. The radar plot and comparative analyses in Figs 12–14 collectively provide a comprehensive visualization and evaluation of SSA’s superior performance in optimizing the allocation and sizing of RDGs within the IEEE 33-bus network.

**Fig 15:**
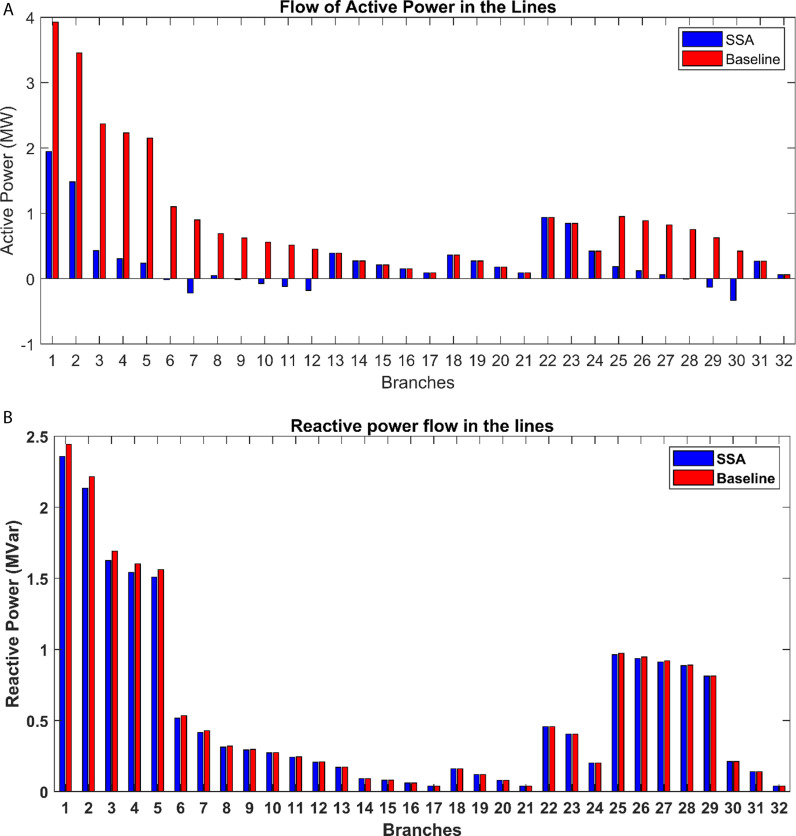
Impact of PV integration on the power flow: (a) active power, (b) reactive power.

**Fig 16:**
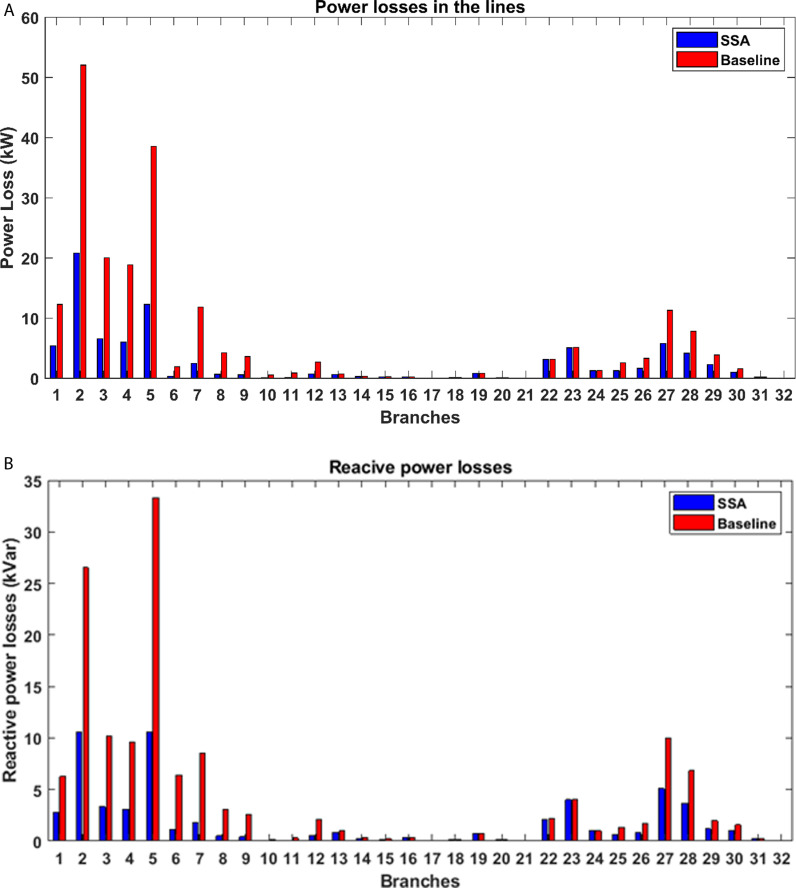
Impact of PV integration on the power losses: (a) active power, (b) reactive power.

The effects of incorporating PV distributed generation into the studied system are depicted in Fig 15. Notably, the alterations in active power flow are most pronounced within the branches adjacent to the buses where PV power plants have been integrated. Fig 16 also illustrates the consequences of introducing PV power plants with optimized capacities at specific locations within the radial distribution network, shedding light on their impact on both active and reactive power losses within the network. Specifically, it becomes evident that there is a substantial drop in real power losses across all branches of the grid thanks to the integration of PV power plants. This reduction in power losses underscores the positive influence of PV integration on network efficiency and reliability. It reflects the potential for PV systems to contribute significantly to the overall power flow dynamics, resulting in enhanced system performance and a more sustainable distribution network.

### B. The results of IEEE 69-node network

**Fig 17:**
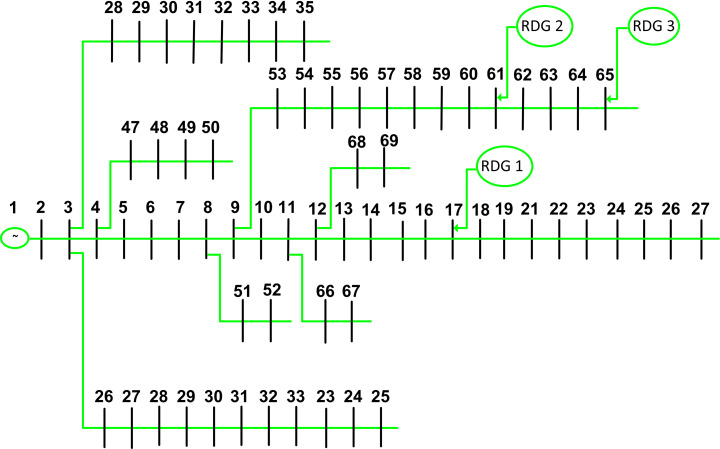
Single line scheme of the IEEE 69-bus network and optimal allocation for three RDG units.

The proposed methodologies underwent rigorous testing on a 69-bus IEEE system with a base voltage set at 12.66 kV and a base MVA adjusted to 100 MVA [35]. The system configuration, depicted in Fig 8, comprises one slack bus and 68 load points, and considers the integration of three RDGs. The optimal placement of these RDGs within the 69-bus test system is visualized in Fig 17. In the absence of RDGs, the 69-bus system exhibits active power losses of 224.963 kW, with the minimum voltage magnitude recorded at 0.9114 per unit (p.u.) at bus 63. Essential information, including Voltage Sensitivity Factor (VSF) and Loss Sensitivity Factor (LSF) values for the IEEE 69-bus system, is presented in Table 4.

**Table 4:** The values of LSF and VSF of the standard IEEE 69-bus system.

69_Bus	LSF	VSF
57	0.0376	0.98976
58	0.01899	0.978016
61	0.01198	0.960285
60	0.00897	0.968139
59	0.00744	0.973474
15	0.00494	1.009806
64	0.00309	0.957548
17	0.00152	1.008304
65	0.000935	0.956938
16	0.00092	1.009239
21	0.000823	1.006971
19	0.000793	1.007801
63	0.0006	0.959565
20	0.00051	1.007484
62	0.00046	0.9599777
25	0.000288	1.006542
24	0.000267	1.0067222
23	0.000123	1.0068883
26	0.000119	1.0064685
27	0. 0000334	1.0064478
18	0. 0000152	1.00829537

The subsequent application of the SSA and other methods, focusing on the first 10 rows of Table 5, reveals noteworthy impacts on power losses and voltage magnitudes with the integration of RDGs (Photovoltaic units) operating at unity power factor. Critical nodes, particularly buses 17, 61, and 65, are identified with significant impacts on the distribution system. The optimal capacities of PV units at these nodes contribute to a total apparent power of 1581.6 KVA at unity power factor. Comparative analyses involving GWO, IGWO, SOA, and MPA demonstrate the superior performance of SSA. With SSA, power losses are reduced from 224.96 kW to 84.3108 kW, marking the highest reduction among the techniques. The minimum voltage magnitude achieved by SSA surpasses that obtained from other algorithms, showcasing a substantial enhancement in the voltage shape. The drop in total power losses corresponds to a significant 62.5221% decrease from the base value, underlining the efficiency of SSA in enhancing the distribution system.

**Table 5:** Obtained results from all techniques for PV system integration(at unity p.f) in IEEE 69- bus.

Results of 69- bus with PV system					
Items	Before optimization	After optimization PV system (at unity PF) for 69-bus			
		GWO	SOA	SSA	MPA
Total losses (kw)	224.9613	77.8119	79.0814	84.3108	82.8439
Loss reduction%	–	65.412%	64.184%	62.5221%	63.174%
V worst (P.u.), bus	0.9114 (63)	0.9724 (61)	0.9715	0.9606 (65)	0.9656
Optimal location and size of DGs (kw)	–	(17) 450 (61) 750 (65) 745	(17) 450 (61) 750 (65) 750	(17) 339.4 (61) 1057.4 (65) 184.8	(17) 450 (61) 642 (65) 750
SDG (KVA)		2100	1950	1581.6	1842
RDGs Power Factor		unity	unity	unity	unity
TOC ($)	–	10811	10064	82424	9248
∆OC		0.9478	0.8823	0.7226	0.8108
∆PlDG		0.3840	0.3869	0.4125	0.4053
∆VD		0.0276	0.0285	0.0394	0.0344

**Fig 18:**
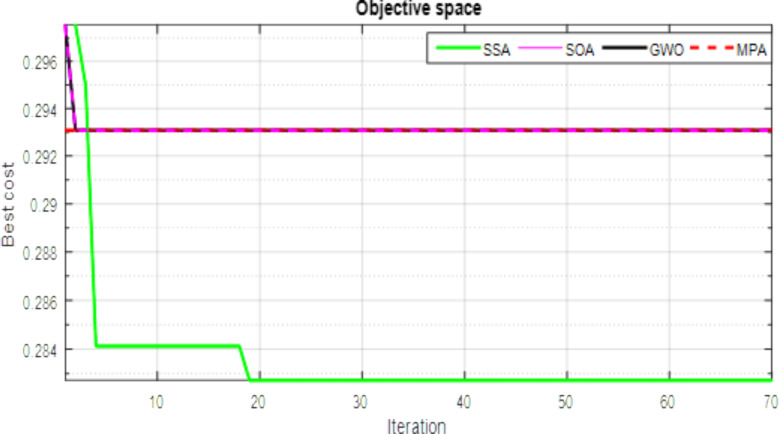
Convergence trends of different methods for IEEE 69-bus test system.

**Fig 19:**
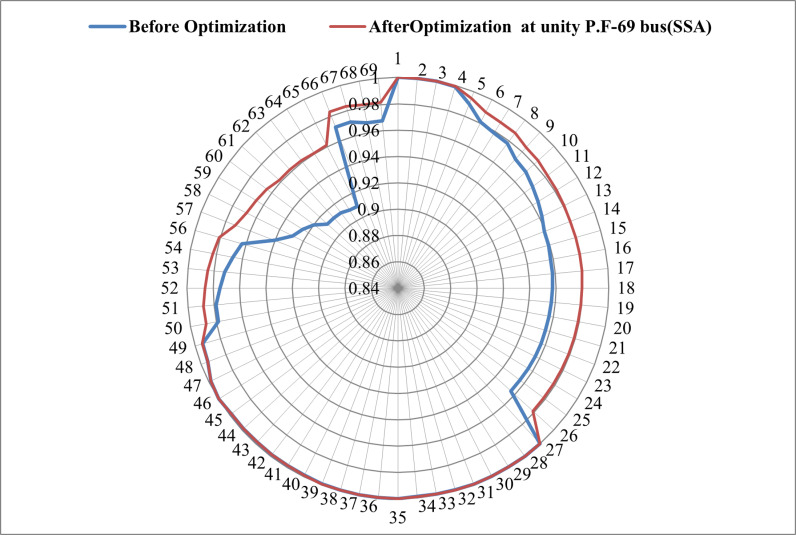
Voltage profile of SSA method after integrating RDG at unity power factor in 69-bus system.

Convergence trends, as illustrated in Fig 18, further emphasize the superiority of salp swarm algorithm in reaching the optimum solution with fewer iterations. Fig 19 vividly portrays the improvement in the voltage shape resulting from the integration of RDG units, with SSA leading in generating the optimal voltage profile.

**Fig 20:**
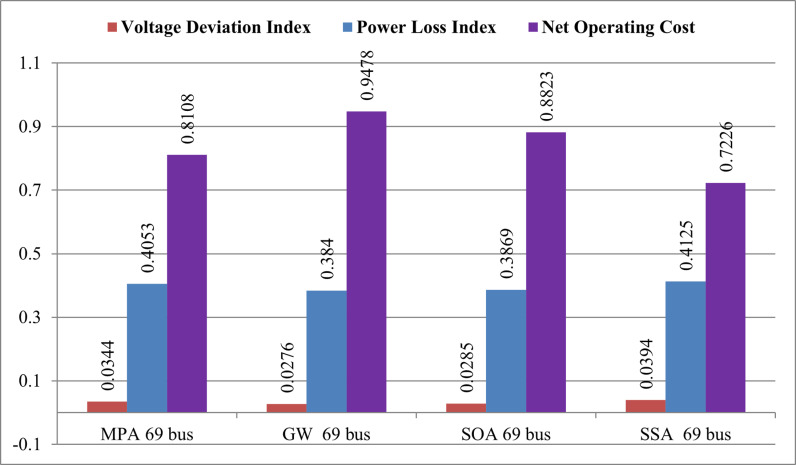
Comparison of different performances for 69-bus system.

A comparative analysis in Fig 20 highlights the favorable performance of SSA, exhibiting significantly reduced operating costs (∆OC) and power loss indices in comparison to MPA, GWO, and SOA. Although the voltage deviation index (∆VD) is comparatively higher, the overall findings from comprehensive analyses, figures, and tables affirm SSA as the most suitable methodology for achieving highest reductions in total power losses and improving the voltage profile in the IEEE 69-bus network.

**Fig 21:**
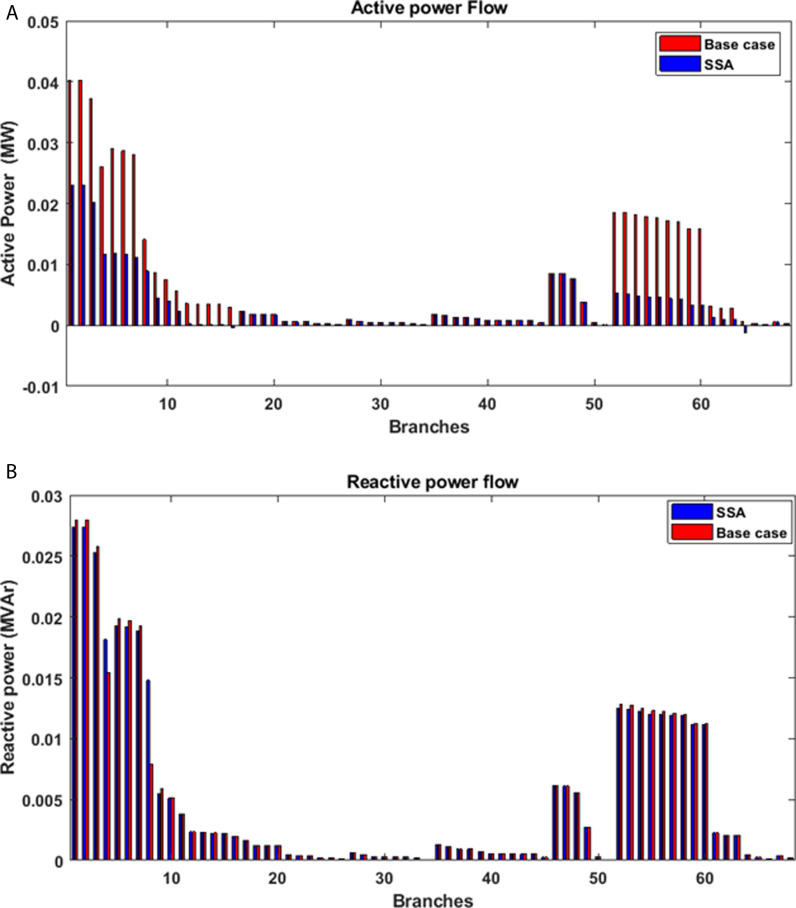
Impact of PV integration into IEEE 69 bus on the power flow: (a) active power, (b) reactive power.

**Fig 22:**
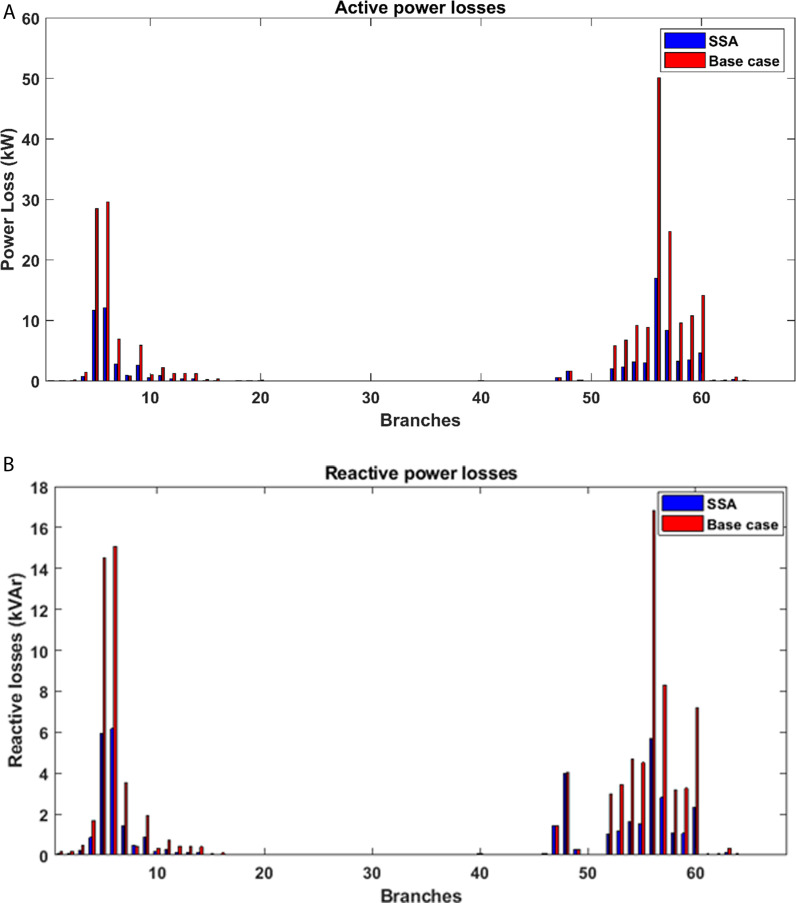
Impact of PV integration into IEEE 69 bus on the power losses: (a) active power, (b) reactive power.

The influence of integrating PV distributed generation into the proposed IEEE 69-bus network is visually represented in Fig 21. Particularly noteworthy are the discernible changes in active power flow, primarily concentrated in the branches adjacent to the buses where PV power plants have been incorporated. Fig 22 further elucidates the impact of strategically placing PV power plants with optimized capacities at specific locations within the radial distribution network. This figure sheds light on the repercussions for both active and reactive power losses within the network. The results distinctly reveal a significant decrease in real power losses across all branches of the system as a consequence of integrating PV power plants. This substantial reduction in power losses underscores the positive impact of PV installation on the efficiency and reliability of the system. It clearly highlights the potential for PV systems to play a crucial role in shaping overall power flow dynamics, contributing to an improved system performance and fostering the development of a more sustainable distribution network.

### C. Case of 118-Bus

**Fig 23:**
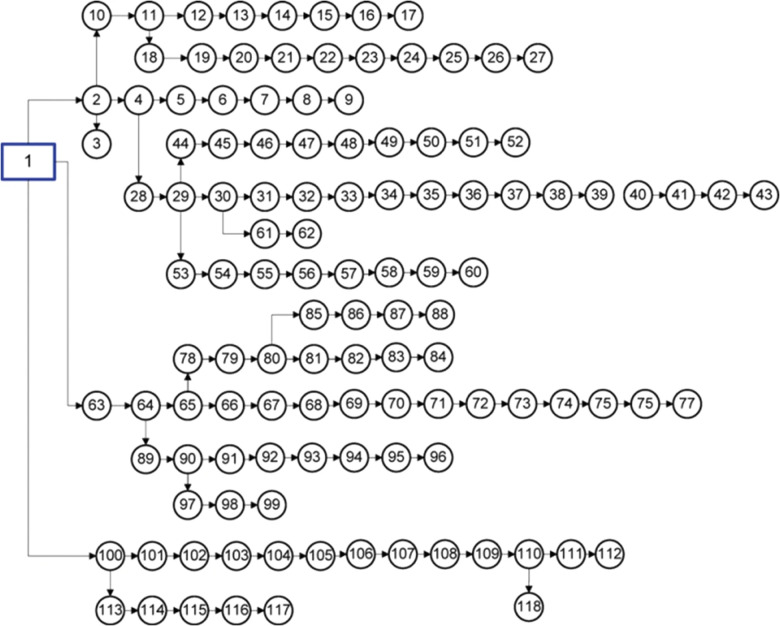
SLD of 118-Bus RDS [ 21].

**Fig 24:**
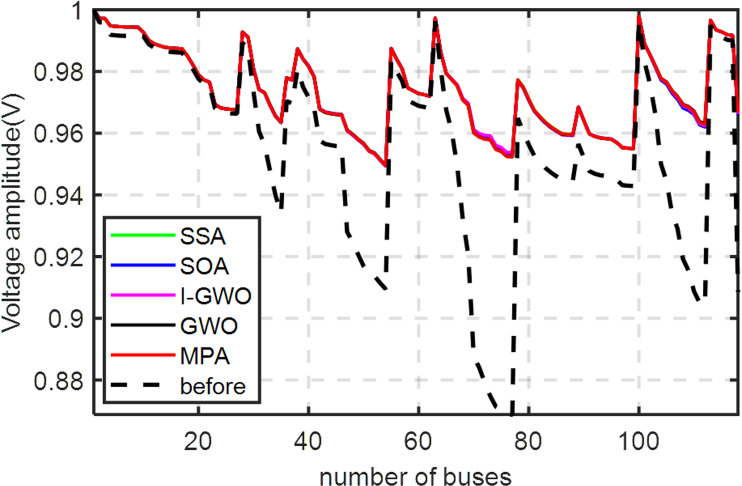
The influence of optimized DGs in the voltage profile for the 118-Bus system.

The single-line diagram (SLD) of the 118-Bus is shown in Fig 23, with the system data detailed in reference [73]. The base values for 118-Bus are set at 100 MVA and 11 kV. Potential DGs buses were pre-identified using both Line sensitivity Factor (LSF) method, as listed in Table 6. A total of 11 buses were selected for optimal DGs allocation by each algorithm. The results from the proposed algorithms, along with other methods, are summarized in Table 7. These results are compared against the MPA and IGWO algorithms, as shown in the table. Fig 24 depicts the impact of DGs on the voltage profile of the 118-b. The results show that the optimized configuration with interconnected DGs can solve the main problem of the 118-bus system of voltage drop. The minimum voltage has been enhanced from 0.868 pu to 0.9494 pu. Moreover, the results of minimization the power loses can be concluded from the table.

**Table 6:** LSF for 118 Bus.

118 Bus	LSF_3_
70	0.031362738770319
104	0.020733329784511
68	0.015510881845002
106	0.014581728425820
108	0.013665938169702
69	0.012612661048411
89	0.012132252568829
67	0.011556752884783
110	0.009984951490346
42	0.008491927394024
47	0.007592467912289
105	0.007252892646407
33	0.007196383172465
34	0.006863179631189
107	0.006716743121693
72	0.005956333070830
35	0.005768012687819
32	0.005755267585559
111	0.005494636442984
73	0.005463628736020
109	0.005431148739767
49	0.005235094580819
50	0.005107744499074
90	0.004660596109736
91	0.004532307646644
48	0.004271583951922
80	0.004260608635861

**Table 7:** The optimized results of the 118-Bus.

Items	Un-Comp.	Compensated									
		MPA		GWO		IGWO		SOA		SSA	
Total losses (kW)	1294.35	586.9490722		587.1186189		587.3078638		587.8807221		586.8148903	
Loss reduction %	–	54.65		54.64		54.63		54.58		54.66	
Vmin (Pu), bus65	0.8688	0.9494		0.9494		0.9494		0.9494		0.9494	
Optimal location buses and size of DGs (kW)	–	32	450.000	32	452.718	32	475.105	32	499.304	32	491.295
		35	954.818	35	972.033	35	949.101	35	910.960	35	921.684
		40	932.094	40	913.280	40	923.886	40	948.754	40	927.863
		50	1750.000	50	1750.000	50	1738.238	50	1750.000	50	1750.000
		70	1150.373	70	1179.623	70	1168.695	70	1179.551	70	1135.019
		73	1434.131	73	1412.698	73	1464.956	73	1431.746	73	1448.168
		79	1384.112	79	1365.738	79	1336.381	79	1345.308	79	1399.586
		105	450.000	105	450.000	105	451.491	105	450.000	105	450.000
		106	450.000	106	450.695	106	452.101	106	450.000	106	450.000
		109	611.060	109	600.236	109	635.804	109	469.300	109	518.899
		110	1453.315	110	1473.321	110	1410.194	110	1535.848	110	1531.835
∑DG, kW	–	11019.9032		11020.34095		11005.95215		10970.77112		11024.34896	
Annual Ploss-cost ($/year)	217450.8	98607.44413		98635.92797		98667.72112		98763.96132		98584.90158	
Total Annual cost ($/year)	217450.8	55099.53949		55101.72821		55029.78423		54853.8791		55121.76826	
Net Saving ($/year)	–	162351.2605		162349.0718		162421.0158		162596.9209		162329.0317	
Saving (%)	–	74.6611466		74.66014		74.6932252		74.7741194		74.6509241	
Pgrid, kW	22709.70	11689.80867		11689.37093		11703.75973		11738.94076		11685.36291	

**Fig 25:**
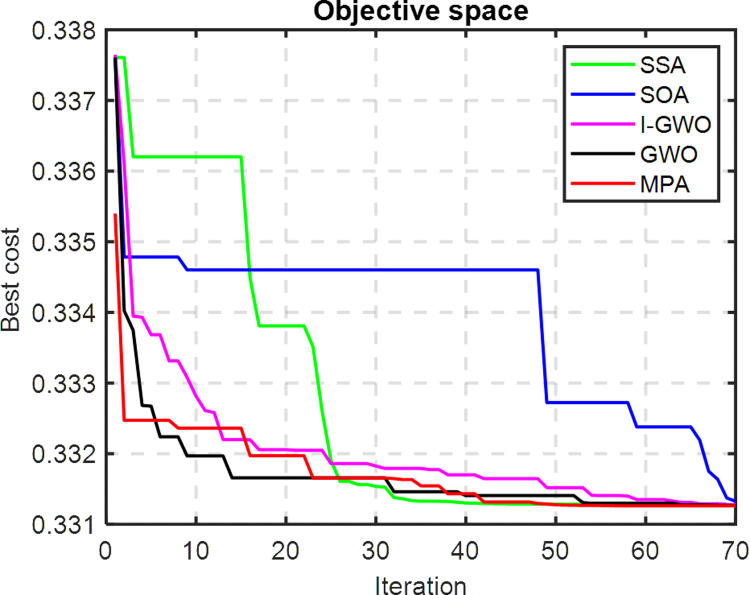
The variation of the objective function with the number of iterations for the 118-bus.

The convergence curve of Fig 25 to show that the 70 iterations (which selected as the remain studied systems in this paper) with the bigger system of 118-bus enhance the results with the superiority of the MPA and SSA. Moreover, it is recommended to increase the number of iterations in the future work.

### D. Real case study, 15 bus system of Middle Egypt Distribution Network

**Fig 26:**
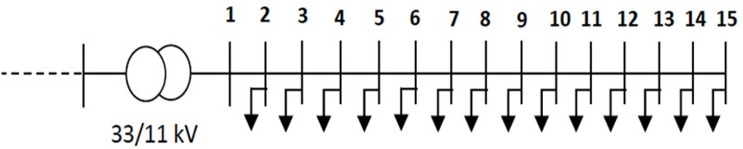
Egyptian case study 15-bus MEDN system.

The system data for the Egyptian case study Middle Egypt Distribution Network (MEDN) used in the calculations are as follows: the base voltage at slack bus 1 is 11kV, the total system apparent power $\left(S_{total}\right)$ is 2201.8+j1018.9kVA, the apparent load power $S_{load}$ is 1976.3+j865.2kVA, and the apparent power losses (S_loss) amount to 225.46+j153.67kVA [46]. The voltage magnitude limits are set between 0.95 and 1.05 p.u. The 15-bus Egyptian MEDN network suffers from poor power quality indices as high voltage drop and losses. The single line diagram of the studied system has been shown in Fig 26.

In this case study, the maximum limit for the total active power $\left(P_{T,DG}^{\text{max}}\right)$ from renewable distributed generators (RDGs) integrated into the MEDN is proposed to be less than the total system load $\left(P_{\text{Load}}\right)$ in order to prevent over-integration of active power into the network. Increasing the maximum limit of integrated active power into the system would naturally enhance the potential for determining the optimal size of RDGs without violating operational constraints.

To validate the effects of RDGs on the MEDN. using the Line Sensitivity Factor (LSF), the optimization algorithms identify buses 7 and 11 as the optimal locations for the RDGs. From Table 8, the optimal capacities for the DGs are 842.72 kW at bus 7 and 543.69 kW at bus 11. As a result, the total system losses are reduced to 81.33%. Additionally, the minimum voltage w$\left(V_{\text{min}}\right)$ is improved from 0.8593 p.u. to 0.957 p.u.

**Table 8:** The optimized results of the Egypt MEDN 15-Bus.

Items	Base Case	MPA		GWO		IGWO		SOA		SSA	
Total losses (kW)	225.46	43.04202238		43.82854572		44.12437958		42.77063761		42.09018212	
Loss reduction %	–	80.9092423		80.5603896		80.4291761		81.0296116		81.3314193	
Vmin (p.u.)/bus	0.8593/15	0.956877574		0.956877574		0.956877574		0.956877574		0.956877574	
Optimal location buses and size of RDGs (kW)	–	(7)	750.00	(7)	740.73	(7)	693.17	(7)	750.00	(7)	842.72
	–	(11)	613.76	(11)	594.99	(11)	642.10	(11)	625.40	(11)	543.69
PDG,T (kW)	–	1363.76		1335.71		1335.27		1375.40		1386.41	
TOC ($)	–	6818.80		6678.56		6676.35		6877.02		6932.06	
Cost of losses	–	7231.06		7363.20		7412.90		7185.47		7071.15	
Cost of DGs	–	6818.80		6678.56		6676.35		6877.02		6932.06	
Pgrid (kW)	2201.8	612.54		640.59		641.03		600.90		589.89	

**Fig 27:**
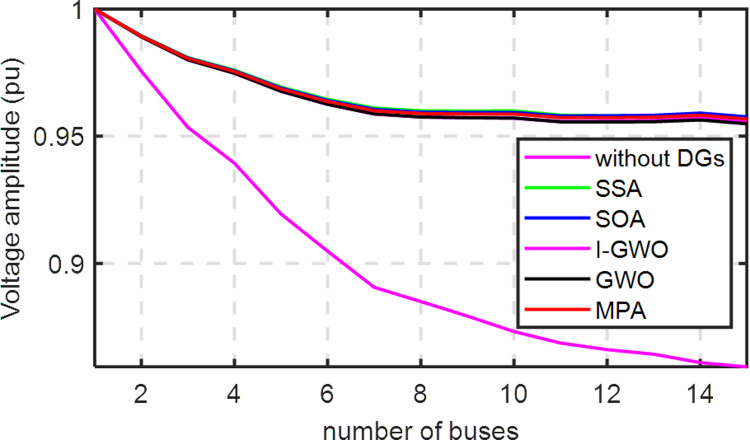
The influence of optimized DGs in the voltage profile for the Egypt MEDN 15-Bus.

**Fig 28:**
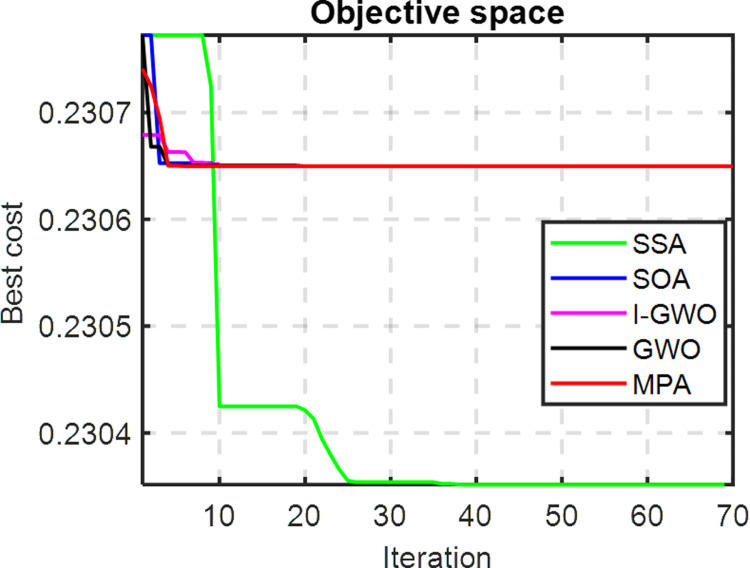
The variation of the objective function with the number of iterations of the Egypt MEDN 15-Bus.

The effects of RDG on the voltage profile of the MEDN is illustrated in Fig 27. These results confirm that the SSA technique is highly efficient, fast, and superior for determining optimal RDG allocations in the MEDN case study. Moreover, one can note that from Fig 28 of the convergence curve of each algorithm.

## V. Statistical results

**Fig 29:**
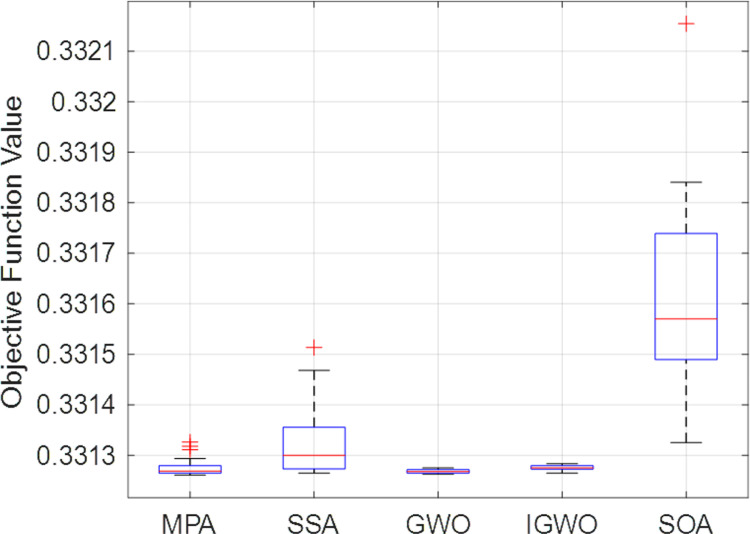
Boxplot comparison of various optimization algorithms for the case of 118 bus system.

To assess the effectiveness of the optimized techniques, it is necessary to establish a set of evaluation metrics based on statistical analysis. Table 9 presents the results of various quality metrics for analyzing the optimization algorithms. These metrics include Relative Error (RE), Mean Absolute Error (MAE), Root Mean Square Error (RMSE), standard deviation (STD), and Median. They evaluate both the optimal minimum value of the objective function that is produced by the optimization algorithms. In Table 8, the number of data set groups of the numbers of individual runs. The case study of 118-bus IEEE system has been considered in this analysis. From this table, it is evident that MPA demonstrates a satisfactory statistical index of min objective function score. Additionally, the STD values indicate that the results remain consistent throughout the iterative process, with values of 0.000004, 0.000005, 0.000020, 0.000072 for GWO, IGWO, MPA and SSA, respectively which showcasing the stability of the algorithms. One can note that the results of all studied algorithms are acceptable. Moreover, Table 9 listed the results of the Wilcoxon signed rank test. The results show that the value is lower than the standard significance level of 5%, a value of h equals 1 which means that the test rejects the null hypothesis, which states that the median is zero. Moreover, for more visualization of the results, Fig 29 shows the boxplot of the values of the objective function via the individual runs.

**Table 9:** Performance metrics comparison of various optimization algorithms for the case of 118 bus system.

	MPA	SSA	GWO	IGWO	SOA
MIN.	0.331261	0.331265	0.331263	0.331265	0.331325
MAX.	0.331326	0.331513	0.331275	0.331283	0.332154
MEAN	0.331277	0.331325	0.331268	0.331276	0.331612
MEDIAN	0.331269	0.331300	0.331268	0.331275	0.331570
STD.	0.000020	0.000072	0.000004	0.000005	0.000194
VARIANCE	1.952579e-05	7.173861e-05	3.89367e-06	4.697291e-06	0.00019388
p_Wilcoxon	0.000089	0.000089	0.000089	0.000089	0.000089
h_Wilcoxon	1	1	1	1	1

## VI. Conclusion

This study introduces the SSA, GWO, IGWO, MPA, and SOA for optimizing the placement and size of RDG units in radial distribution networks. Additionally, sensitivity factors are incorpoated to identify key candidate buses crucial for achieving the optimal allocation of RDGs in distribution networks. The efficacy of the proposed optimization technique is demonstrated through its application on 33-bus and 69-bus IEEE RDS under various scenarios. In addition, a larger RDS system, the 118-bus IEEE system, was analyzed to improve its power quality indices. Furthermore, a real-world case study involving a 15-bus system from Egypt was considered and evaluated using the applied techniques. Furthermore, the results obtained from SSA are compared with those from other algorithms to validate its effectiveness. The findings underscore that the SSA technique is not only exceptionally fast but also outperforms other widely used methods in identifying the optimal placement and sizing of RDG units in distribution networks. The proposed technique aims to enhance the voltage profile, minimize total power losses, and reduce overall operational costs for RDG units. In the 33-bus system operating at unity Power Factor (PV system), SSA achieves the optimal solution within 64 seconds, while the MPA algorithm requires 114 seconds for the same task. Furthermore, with the SSA technique, the total active power losses in the system are reduced to 84.7475 kW, and the lowest voltage magnitude is raised to 0.9637 p.u. The total operating cost (TOC) decreased to $9597.1. In comparison, the MPA approach reduces total power losses to 84.7803 kW, decreases TOC to $9583.5, and raises the lowest voltage magnitude to 0.9636 p.u. These results affirm that the suggested SSA methodology is proficient in minimizing total operating costs, minimizing real power losses, and enhancing the voltage shape compared to other techniques. As for future works, the robustness of studied optimization algorithms and other hybrid optimization techniques in handling uncertainties or dynamic scenarios could be explored for a more comprehensive understanding of its applicability in real-world distribution systems.

## Supporting information

S1 File Supporting information. (ZIP)

## Abbreviations

WT: Wind turbines
PV: Photovoltaic
DG: Distributed generation
RDGs: Renewable distributed generations
MPA: Marine Predictor Algorithm
SSA: Salp Swarm Algorithm
GWO: Grey Wolf Optimizer
IGWO: Improved Grey Wolf Optimizer
SOA: Seagull Optimization Algorithm
RDS: Radial distributed systems
EA: Efficient analytical
ABC: Artificial bee colony method
PPA: Plant propagation algorithm
MRFO: Manta Ray Foraging optimization algorithm
HBA: Honey badger algorithm
MLPSO: Multileader particle swarm optimization
CapSA: Capuchin search algorithm
HBMO: Honey bee mating optimization
WOA: Whale optimization algorithm
EO: Equilibrium optimization
HHO: Harris hawks optimizer
MVO: Multi Verse Optimization
TSA: Tunicate Swarm Algorithm
HS: harmony search
SOA: Seagull Optimization Algorithm
DLH: Dimension learning-based hunting
∆P_1,DG_: Power Losses Index
OF: Objective function
${\text{P}}_{\text{loss}}^{\text{T}}$: Total real power losses
MCS: Modified crow search
ACS: Adaptive cuckoo search
MGBO: Modified Gradient-Based Optimization
FA: Fireworks algorithm
MPGS: Modified plant growth simulation
JS: Jellyfish search algorithm
IAVO: Improved African vultures optimizer
AGTO: Artificial Gorilla Troops Optimizer
AEO: Artificial ecosystem- based optimization
GOA: Grasshopper Optimization Algorithm
CS: Cuckoo Search
IPSO–GSA: Improved PSO and gravitational search algorithm
ESFO: Enhanced sunflower optimization
MFO: Moth-flame optimization
LSFs: Loss sensitivity factors
BFOA: Bacterial foraging optimization algorithm
BSOA: Backtracking search optimization algorithm
MOSCA: Multi-objective sine cosine algorithm
RDN: Radial Distribution Networks
BFS: Forward–backward sweep
LSF: Loss Sensitivity Factor
VSI: Voltage stability index
TOC: Total Operational Cost
VSF: Voltage Sensitivity Factors
TOC: Total ownership cost
∆VD: Indices for voltage deviation
∆OC: Operating cost deviation
∆V_Dev_: Voltage deviation index
n_br_: Number of the branches
