# Supplementary material for: Various optimization algorithms for efficient placement and sizing of photovoltaic distributed generations in different networks
Source: PLoS One. 2025 Apr 2;20(4):e0319422. doi: 10.1371/journal.pone.0319422 (PMC11964283; doi:10.1371/journal.pone.0319422)
Supplement: S1 File — (ZIP) [file pone.0319422.s001.zip › Supporting information.docx]

Supporting information

Various optimization algorithms for efficient placement and sizing of photovoltaic distributed generations in different networks

The data set of three test distribution networks can be found as:
IEEE 33-bus system.xlsx

IEEE 69-bus system.xlsx

data118bus_system.txt
